# Does osteogenic potential of clonal human bone marrow mesenchymal stem/stromal cells correlate with their vascular supportive ability?

**DOI:** 10.1186/s13287-018-1095-7

**Published:** 2018-12-19

**Authors:** Alison T. Merryweather-Clarke, David Cook, Barbara Joo Lara, Peng Hua, Emmanouela Repapi, Neil Ashley, Shiang Y. Lim, Suzanne M. Watt

**Affiliations:** 1 Stem Cell Research, Nuffield Division of Clinical Laboratory Medicine, Radcliffe Department of Medicine, University of Oxford, John Radcliffe Hospital, Oxford, OX3 9BQ UK; 2 Stem Cell Research, NHS Blood and Transplant, John Radcliffe Hospital, Oxford, OX3 9BQ UK; 3 Weatherall Institute of Molecular Medicine, Radcliffe Department of Medicine, John Radcliffe Hospital, Oxford, OX3 9BQ UK; 4 Department of Surgery, University of Melbourne, Fitzroy, Victoria 3065 Australia; 5 O’Brien Institute Department, St. Vincent’s Institute of Medical Research, Fitzroy, Victoria 3065 Australia

**Keywords:** Mesenchymal stem/stromal cells, Osteogenesis, Vascular supportive capacity, RNAseq, Clonal analysis, CFU-F

## Abstract

**Background:**

Human bone marrow-derived mesenchymal stem/stromal cells (hBM MSCs) have multiple functions, critical for skeletal formation and function. Their functional heterogeneity, however, represents a major challenge for their isolation and in developing potency and release assays to predict their functionality prior to transplantation. Additionally, potency, biomarker profiles and defining mechanisms of action in a particular clinical setting are increasing requirements of Regulatory Agencies for release of hBM MSCs as Advanced Therapy Medicinal Products for cellular therapies. Since the healing of bone fractures depends on the coupling of new blood vessel formation with osteogenesis, we hypothesised that a correlation between the osteogenic and vascular supportive potential of individual hBM MSC-derived CFU-F (colony forming unit-fibroblastoid) clones might exist.

**Methods:**

We tested this by assessing the lineage (i.e. adipogenic (A), osteogenic (O) and/or chondrogenic (C)) potential of individual hBM MSC-derived CFU-F clones and determining if their osteogenic (O) potential correlated with their vascular supportive profile in vitro using lineage differentiation assays, endothelial-hBM MSC vascular co-culture assays and transcriptomic (RNAseq) analyses.

**Results:**

Our results demonstrate that the majority of CFU-F (95%) possessed tri-lineage, bi-lineage or uni-lineage osteogenic capacity, with 64% of the CFU-F exhibiting tri-lineage AOC potential. We found a correlation between the osteogenic and vascular tubule supportive activity of CFU-F clones, with the strength of this association being donor dependent. RNAseq of individual clones defined gene fingerprints relevant to this correlation.

**Conclusions:**

This study identified a donor-dependent correlation between osteogenic and vascular supportive potential of hBM MSCs and important gene signatures that support these functions that are relevant to their bone regenerative properties.

**Electronic supplementary material:**

The online version of this article (10.1186/s13287-018-1095-7) contains supplementary material, which is available to authorized users.

## Background

Mesenchymal stem and stromal cells (MSCs), also known as mesenchymal or skeletal stem and progenitor cells (MSPCs or SSCs), are critical components of the post-natal bone marrow (BM). When derived from this tissue, they have the potential not only to differentiate into multiple lineages (including osteogenic (O), chondrogenic (C) and adipogenic (A)), but also to form niche cells which support haematopoietic and blood vessel formation and function (reviewed in [1–7]). Additionally, when transplanted, these cells can display significant immunosuppressive, anti-inflammatory and anti-fibrotic properties (reviewed in [8–13]). These unique features of hBM MSCs have been key to their development from in vitro and in vivo pre-clinical models to numerous clinical trials, where they have, for example, been used to treat bone and joint disorders, improve haematopoietic stem cell engraftment, expand haematopoietic stem and progenitor cells, enhance blood vessel formation, ameliorate auto-immune disorders and graft versus host disease, and prevent or reduce fibrosis and scarring (reviewed in [5–7, 14–16]).

Historically, MSCs have been notoriously poorly defined. They were initially characterised by their clonogenic colony-forming unit-fibroblast (CFU-F) formation [17–19], adherence to plastic, expression of characteristic combinations of stromal cell markers (e.g. CD90, CD105, CD73) while being CD45 and CD31 negative), and their multi-lineage differentiation capacity [20, 21]. Muraglia et al. [22, 23] examined the differentiation capacity of single hBM derived CFU-F clones over time and reported heterogeneity of their potentiality, with 99% of hBM CFU-F clones having osteogenic potential, of which approximately one third possessed tri-lineage AOC potential and 60–80% OC potential. They found no clones with OA or CA potential, but demonstrated that tri- and bi-potent clones progressively lost their adipogenic and chondrogenic potential with increasing hBM MSC population doublings. Various other reports indicate that 20–50% of clonal CFU-F from non-enriched human bone marrow have at least tri-lineage AOC potential ([24] and references therein). These studies highlight the importance, amongst other parameters, of donor age, source of cells (e.g. iliac crest, bone fragments), culture conditions, passage number and the pre-separation protocols and isolation markers used to analyse these cells. As an exemplar, our own unpublished studies [Watt SM, unpublished data] and studies from others [25–27] demonstrate a significant loss of hBM MSCs if bone marrow is fractionated on density gradients which select cells with densities < 1.077 g/ml. Thus, both plastic adherent CFU-F and those derived after enrichment on density gradients and with the a variety of cell surface markers (e.g. CD271, CD146, MSCA-1, CD105, STRO-1, PDGFR-β (CD140b), ErbB2 (CD340), Frizzled-9 (CD349), CD90, CD295 (LepR), CD106, VCAM-1 CD140a^low/−^) [28–44] possess inherent and/or cell selection biased heterogeneity, and contain both multipotent MSCs and partially differentiated mesenchymal progenitor cells. This functional heterogeneity of hBM MSCs represents a major challenge in developing isolation, and potency and release assays, which can sustain and predict their functionality prior to transplantation [5, 45, 46].

Our previous studies have demonstrated the hBM MSCs not only differentiate into osteogenic, adipogenic and chondrogenic lineages, but can also support vascular tubule formation [47–50]. We have shown that this vasculogenic support can be mimicked with a clonal nestin^+^ murine bone marrow MSC cell line (MS-5) which also supports human haematopoiesis [51–54]. In this manuscript, we first defined the CFU-F-derived clonal populations of hBM MSCs by their AOC tri-lineage and more restricted lineage differentiation potentials from erythrocyte-depleted whole human bone marrow from young donors (< 40 years). Since the healing of bone defects in larger bone grafts or fractures depends on the coupling of new blood vessel formation with osteogenesis [55–59], we examined the potential of these individual hBM MSC clones, with differential osteogenic differentiation capacities, to support endothelial networks in co-culture assays in vitro. Next, we studied the transcriptome of a proportion of clonal CFU-F cultures, which show a range of osteogenic and endothelial supportive capacities, using RNAseq to better characterise the gene expression profile of the clonally derived cells with different lineage potentials and to assess their differential expression of factors important and potentially predictive for their capacity for osteogenic-angiogenic coupling.

## Methods

### Human bone marrow preparation and culture

Fresh human bone marrow aspirates (30 ml) from the iliac crest of healthy adult donors less than 40 years of age were purchased from Lonza, Slough, England. To avoid the loss of hBM MSCs on density (< 1.077 g/ml) gradients [25–27], red blood cells were lysed by adding three parts red cell lysis buffer (150 mM ammonium chloride, 10 mM potassium bicarbonate, 0.1 mM EDTA) to one part bone marrow aspirate for 10 min at room temperature with gentle agitation. Samples were washed twice by centrifugation at 350 g for 10 min in PBS (Lonza) prior to resuspension in mesenchymal stem cell growth media (MSCGM; Lonza). The cells were then passed through a 70-μm cell strainer, counted using trypan blue to exclude dead cells and seeded directly onto tissue culture plastic flasks for expansion. Seeding densities varied depending on the assay. For standard bulk cell expansion, red blood cell-depleted bone marrow cells were seeded at approximately 5 × 10^5^/cm^2^ in MSCGM (Lonza) in tissue culture grade plastic dishes or flasks, the cells allowed to adhere for 48 h before full replacement of the MSCGM (Lonza). The hBM MSCs were cultured in MSCGM (Lonza) with media changes every 3–4 days and passaged with trypsin/EDTA when approximately 90% confluent.

### Colony forming unit-fibroblast (CFU-F) quantitation

Red blood cell depleted fresh human bone marrow cells were counted and seeded at 1.25 × 10^4^, 2.5 × 10^4^ and between 1 × 10^5^ to 5 × 10^4^ cells/cm^2^ into 6 well plates or 10 cm diameter tissue culture plastic dishes (Corning Life Sciences, Tewksbury, MA, USA) in MSCGM (Lonza). The media were changed every 3–4 days and cells cultured for approximately 2 weeks or until discrete colonies could be identified under the microscope. The colonies were washed twice in PBS and fixed in 95% ethanol for 5 min. The ethanol was removed and the cells stained for 30 min in 0.5% crystal violet in 95% ethanol. The crystal violet was removed, and the plates washed in tap water to remove excess stain. The plates were then allowed to dry before CFU-F imaging and counting using the LICOR Odyssey system (Lincoln, NE, USA).

### Generation of clonal cell cultures

Bone marrow cells were seeded as a single-cell suspension into 10 cm diameter tissue culture plastic dishes at 1.25 × 10^4^, 2.5 × 10^4^ and 5 × 10^4^ cells/cm^2^ in MSCGM (Lonza). The cells were cultured as described above for approximately 2 weeks, until large colonies formed. At this stage, single colonies were picked using cloning cylinders, and the cells from these individual colonies transferred to individual wells of 12 well plates for further expansion. At 90% confluency (passage (P) 0), the clonal cultures were passaged into T25 flasks. When the cultures reached 90% confluency (termed P1), they were used at this passage in the AOC differentiation and vascular tubule co-culture assays as described below. Typical scoring of the cultures in these differentiation assays is illustrated in Additional file 1: Figure S1.

### Differentiation assays

For the differentiation assays, hBM MSCs were seeded at 2 × 10^4^ cells/cm^2^ into 96 well plates in MSCGM (Lonza) and allowed to attach overnight. Collagen I-coated plates were used for osteogenic and chondrogenic cultures. The medium was then replaced with differentiation medium (Lonza) to promote osteogenic, adipogenic and chondrogenic differentiation. Osteogenic differentiation media comprised osteogenic basal media plus hMSC osteogenic Singlequot supplements and growth factors (dexamethasone, ascorbate, mesenchymal cell growth supplement (MCGS), l-glutamine, penicillin/streptomycin, β-glycerophosphate). Adipogenic differentiation media comprised adipogenic induction media plus hMSC adipogenic induction Singlequot supplements and growth factors (h-Insulin (recombinant), l-glutamine, mesenchymal cell growth supplement (MCGS), GA1000, dexamethasone, indomethacin and IBMX). Chondrogenic differentiation media comprised chondrogenic differentiation media plus hMSC chondrogenic Singlequot supplements and growth factors (ITS + supplement, dexamethasone, ascorbate, sodium pyruvate, proline, GA-1000, l-glutamine). These media were replaced every 3–4 days as described in the manufacturer’s protocol. Adipogenic and chondrogenic potentials were assayed at 14 days, while osteogenic differentiation was assayed at 21 days.

### Oil Red O staining

For adipogenic differentiation, lipid droplets were visualised using Oil Red O staining. Cells were washed with PBS, fixed with 4% paraformaldehyde (PFA) for 10 min, washed once in double distilled water and incubated in 60% isopropanol for 5 min. After isopropanol removal, cells were incubated with Oil Red O staining solution (0.3% Oil Red O in 60% isopropanol) for 10 mins, and washed once in 60% isopropanol and then 3 times with double distilled water. The samples were stored in 20% glycerol in PBS and imaged using bright field microscopy. For quantification, Oil Red O was extracted using 100% isopropanol (50 μl/well) for 10 min and the absorbance (490 nm) measured using a Biorad plate reader.

### Alizarin Red staining for osteogenic differentiation

Calcification of osteogenic monolayers was visualised using Alizarin Red staining. Cells were washed with PBS, fixed with 4% PFA for 15 min and washed three times in PBS and once with double distilled water before staining with 40 mM Alizarin Red S solution (pH 4.2) for 20 min at room temperature. The cells were washed in double distilled water and plates dried prior to imaging. For quantification, Alizarin Red S was extracted by incubating the wells in 150 μl 10% acetic acid for 30 min. The sample was then transferred to a 96-well qPCR plate and incubated at 85 °C for 10 min followed by incubation on ice for 5 min. One hundred μl of sample was then transferred to a new 96 well plate, and the pH was neutralised with 30 μl 10% ammonium hydroxide. The absorbance was then measured at 415 nm using a Biorad plate reader.

### Alcian blue staining for chondrogenic differentiation

The cells were washed with PBS and fixed with 100% ice cold methanol for 10 min. The cells were washed three times in PBS and once with 0.1 M HCl before staining with Alcian blue staining solution (1% Alcian blue, 0.1 M HCl) for 30 min at room temperature (RT). The cells were then washed once with 0.1 M HCl and three times with double distilled water. Plates were allowed to dry prior to imaging.

### Vascular tubule co-culture assay

HUVEC cultures (P1) were purchased from Lonza, cultured in EGM-2 (Lonza) with media changes every 2–3 days and passaged at 95% confluency with trypsin/EDTA. hBM MSCs were seeded at 2 × 10^4^ and 4 × 10^4^ cells/cm^2^ into 96-well plates and incubated overnight in MSCGM (Lonza). The next day the media were removed and HUVECs at passage 3–5 were added to the hBM MSCs at a concentration of 2.5 × 10^3^ cells/cm^2^ in EGM2. The co-cultures were fed every 2 to 3 days with complete EGM-2 (Lonza) [48]. After 14 days’ incubation, the cells were fixed in ice-cold 70% (*v*/*v*) ethanol for 30 min at RT and washed three times with PBS. For vessel staining, the PBS was removed and cells were incubated for 30 min with 50 μl of blocking buffer (5% (*w*/*v*) BSA in PBS) at room temperature and then with mouse anti-hCD31 primary antibody (1:4000) in blocking buffer (AbD Serotec, Kidlington, England) overnight at 4 °C followed by 50 μl of biotinylated-goat anti-mouse Ig (1:200) (Vector Laboratories, Peterborough, England) at RT for 1 h. After three washes with PBS, 50 μl of Vectastain Elite ABC reagent and then 50 μl of DAB Peroxidase Substrate working solution (both from Vector Laboratories) for 10 min were applied. Subsequently, the cells were washed three times with double distilled water for 5 min and allowed to dry overnight before imaging and quantification. In order to quantify the tubules, images were taken with a × 4 objective on a NIKON Eclipse TE2000-U microscope (London, England) and processed in Adobe Photoshop CS2 version 9.0 (Adobe Systems Inc., Maidenhead, England). To quantify tubule formation, the images were analysed using Angiosys 1.0 software (Cellworks, Buckingham, England).

### Flow cytometry

The hBM MSCs at P1 were characterised by flow cytometry as described previously [48] using antibodies described in Additional file 2: Table S1 and analysed using a BD LSRII flow cytometer. Data were collected by FACS DIVA Software (Version 6.13; firmware version 1.9; Becton-Dickinson, Oxford, England) and analysed with FlowJo software (Ashland, OR, USA). The cells were confirmed for their MSC phenotype as CD45^−^ and CD73^+^CD105^+^CD90^+^ CD166^+^CD146^+^.

### Statistics for cellular studies

Statistical analyses were performed using GraphPad Prism version 7 (Graphpad Software Inc., San Diego, CA, USA). *P* values < 0.05 were considered statistically significant. Data are presented as means ± SD for the number of biological replicates indicated in the “Results” section. Pearson’s correlation coefficient (*r*) was used to measure the strength of the association between the vascular tubule supportive and osteogenic or adipogenic potential, or between the osteogenic and adipogenic potential of the CFU-F clones from human bone marrow.

### hBM MSC sorting and cDNA library generation

The hBM MSC clonal cultures, cryopreserved at the time of the differentiation and vascular tubule support analyses (P1), were thawed and diluted in warm MSCGM (Lonza) before centrifugation at 300 g and resuspended in FACS buffer. DAPI (Sigma-Aldrich Ltd., St Louis, MI, USA) was added as a viability stain before flow cytometric sorting. One hundred single live cells (DAPI positive cells excluded) for each clonal culture being analysed at P1 were sorted into PCR tubes containing lysis buffer, oligo-dT primer and dNTP mix, and Smart-Seq2 cDNA libraries were then generated as described [60].

### RNA sequencing and analysis

Sequencing was performed using TruSeq SBS Kit v3 chemistry (Illumina Inc., Cambridge, England). Following QC analysis with the fastQC package (http://www.bioinformatics.babraham.ac.uk/projects/fastqc), reads were aligned using STAR [61] against the human genome assembly (NCBI build37 (hg19) UCSC transcripts). Non-uniquely mapped reads were discarded using SAMtools [62]. Gene expression levels were quantified as read counts using the featureCounts function [63] from the Subread package [64] with default parameters. The read counts were used for the identification of global differential gene expression between specified populations using the edgeR package [65]. RPKM (reads per kilobase of transcript per million mapped reads) values were also generated using the edgeR package. Genes were considered differentially expressed between populations if they had an adjusted *p* value (FDR, false discovery rate) of less than 0.05 (high stringency) or 0.1 (relaxed stringency). Heat maps and clustering of RNA expression were generated using Genesis [66].

### Enrichment analysis

MetaCore (Thompson Reuters, London, England) was used to determine pathways enriched in genes in the upper expression quartile and in genes differentially expressed between different groups of hBM MSC clones. We used Venny [67] to construct Venn diagrams displaying numbers of transcripts identified as expressed by both hBM MSCs described in this manuscript (termed UoOX hBM MSCs) and also W8B2+ hBM MSCs purified and published by Zhang et al. [68]. Boxplots constructed using GraphPad Prism version 7 (Graphpad Software Inc., San Diego, CA, USA) displayed expression levels, as log RPKM values for UoOX hBM MSCs and log FPKM values for W8B2+ hBM MSCs, of all transcripts, shared transcripts and unique transcripts in each data set.

### Quantitative real-time PCR

RNA was isolated from one million cryopreserved cells selected from higher versus lower osteogenic CFU-F clones (*n* = 6) at P1 using RNeasy Plus Mini Kit (QIAGEN, Valencia, CA, USA) according to the manufacturer’s protocol. RNA concentrations were determined using NanoDrop One (ThermoFisher, Loughborough, England) then each RNA sample was assayed twice and the mean value taken. cDNA synthesis was carried out by reverse transcription of total RNA to cDNA using the High Capacity RNA-to-cDNA kit (Applied Biosystems, ThermoFisher) following the protocol recommended by the manufacturers. Five (5) nanograms of cDNA was used in each qPCR reaction on the QuantStudio 7 Flex System (ThermoFisher) supplied with Power SYBR Green PCR Master Mix (Applied Biosystems, ThermoFisher) and primers (custom synthesised by Integrated DNA Technologies, Coralville, IA, USA) as listed below. All these genes were analysed at least three times. The relative expression level of each gene was calculated using ΔΔCt method against GAPDH. The Student *T* test was used for statistical comparison of each gene and *p* < 0.05 taken as statistically significant.

**Table:** 

Gene	Primers
*GAPDH*	GTTCGACAGTCAGCCGCATC GGAATTTGCCATGGGTGGA
*SPARC*	TGCCTGATGAGACAGAGGTGGT CTTCGGTTTCCTCTGCACCATC
*BAD*	GAGCCCGGGGTGCTGGAGGGA GGCGGCACAGACGCGGGCTTT
*CXCL12*	TCAGCCTGAGCTACAGATGC CTTTAGCTTCGGGTCAATGC
*EGLN1*	TGAGCAGCATGGACGACCTGAT CGTACATAACCCGTTCCATTGCC
*GRB14*	TACCCAGTGACATAACGGCTCG GCACTTCAATCACCAGTTCGTGG
*COL12A1*	GTCCCAGGATGAGGTCAAGA TGGCAAGCTCATTGTAGTCG

## Results

### Generation and potency of hBM MSC-derived CFU-F

Thirty milliliters of each fresh human bone marrow aspirate were red blood cell depleted and the total nucleated cell (TNC) counts determined. These ranged from 2.2 × 10^8^ to 3.8 × 10^8^ (mean + SD = 2.82 + 0.86 × 10^8^; *n* = 3) TNC per 30 ml bone marrow aspirate. The CFU-F content of these cells was next assessed and this ranged from 1 in 16,000 to 1 in 33,500 bone marrow cells (equivalent to 37.25 + 4.60 CFU-F per 5 × 10^8^ red cell lysed human bone marrow TNCs; Table 1; Additional file 3: Figure S2).

**Table 1:** CFU-F content in human bone marrow aspirates

Bone marrow donor	Cell number after red cell lysis	CFU-F number from 4.8 × 10^5^ cells	Frequency of CFU-F per bone marrow aspirate
1	3.80 × 10^8^	30	1/16,000
2	2.48 × 10^8^	20	1/24,000
3	2.19 × 10^8^	14	1/33,500

Since therapeutic doses of hBM MSCs require their expansion in vitro, we isolated a total of 133 CFU-F colonies (P0) and expanded these into T25 tissue culture grade flasks (P1). All 133 clones were analysed at P1 for their morphology, ability to differentiate into osteoblasts, adipocytes and chondrocyte*s* in vitro, and for their vascular tubule support in co-culture assays with HUVEC. Osteogenic and adipogenic differentiation was also quantified colorimetrically relative to a control non CFU-F selected hBM MSC (C that had been run in parallel for all assays in order to minimise the effects of performing multiple assays. Representative images of the morphology and comparative confluency of hBM MSCs derived from the individual CFU-Fs are shown in Additional files 4 and 5: Figures S3 and S4. Although they exhibited a typical spindle-shaped fibroblastoid appearance, the proliferative ability and functional potency in vitro of each clone varied in culture. All three bone marrow aspirates produced CFU-F with a range of AOC differentiation capacities. Of the total 133 clones, 95% possessed osteogenic potential, (AOC, OC, OA, O), 64% demonstrated tri-lineage AOC potential, 31% were bi-potent (OA, AC, OC), while only 4% were unipotent (O or C) and 1% nullipotent (Table 2). No clones were detected with adipogenic (A) potential only. These differed slightly from studies Muraglia et al. [22] and Alessio et al. [69]. In agreement with our data, these other studies showed that the percentage of AOC and OC clones and of clones possessing osteogenic potential predominated in adult human bone marrow, although Muraglia et al. [22] also demonstrated that their AOC versus OC frequency and an increase in unipotent osteogenic clones (O) was donor age dependent. However, while we detected a small proportion of clones with AC, OA, C or O potential (with the exception of osteogenic potential in older donors), Muraglia et al. [22] did not detect these, while Alessio et al. [69] detected all clone types (AOC, OC, OA, AC, C, O, A). These differences may be due to different growth media usage and different donor characteristics but suggest the existence of CFU-F clones where osteogenic, adipogenic and chondrogenic potential can be combined independently.

**Table 2:** The differentiation capacity of CFU-F clones from human bone marrow

(A)					
Lineage potential	% of total clones (Number of clones)	% of upper quartile, Tubule Length (Number)	% of lower quartile, Tubule Length (Number)	% of clones within group that fall within Tubule Length Upper quartile	% of clones within group that fall within Tubule Length Lower quartile
AOC	64% (85)	73% (24)	50% (17)	29%	20%
OC	24% (32)	24% (8)	29% (10)	25%	31%
AO	5% (6)	0%	3% (1)	0%	17%
AC	2% (3)	0%	3% (1)	0%	33%
O	2% (3)	3% (1)	6% (2)	33%	67%
C	2% (3)	0%	6% (2)	0%	67%
Null	1% (1)	0%	3% (1)	0%	100%
(B)					
Lineage potential	% of total clones (Number of clones)	% of upper quartile, Osteogenesis Alizarin Red (Number)	% of lower quartile, Osteogenesis Alizarin Red (Number)	% of clones within group that fall within Osteogenesis Alizarin Red Upper quartile	% of clones within group that fall within Osteogenesis Alizarin Red Lower quartile
AOC	64% (85)	70% (23)	29% (10)	27%	11%
OC	24% (32)	21% (7)	38% (13)	22%	41%
AO	5% (6)	9% (3)	6% (2)	50%	33%
AC	2% (3)	0%	9% (3)	0%	100%
O	2% (3)	0%	6% (2)	0%	67%
C	2% (3)	0%	9% (3)	0%	100%
Null	1% (1)	0%	3% (1)	0%	100%
(C)					
Lineage potential	% of total clones (Number of clones)	% of upper quartile, Adipogenesis Oil Red O (Number)	% of lower quartile, Adipogenesis Oil Red O (Number)	% of clones within group that fall within Adipogenesis Oil Red O Upper quartile	% of clones within group that fall within Adipogenesis Oil Red O Lower quartile
AOC	64% (85)	91% (30)	26% (9)	35%	11%
OC	24% (32)	3% (1)	62% (21)	3%	66%
AO	5% (6)	3% (1)	3% (1)	17%	17%
AC	2% (3)	3% (1)	0%	33%	0%
O	2% (3)	0%	3% (1)	0%	33%
C	2% (3)	0%	3% (1)	0%	33%
Null	1% (1)	0%	3% (1)	0%	100%

The numbers of tripotent (AOC with adipogenic, osteogenic and chondrogenic potential), bipotent (OC with osteogenic and chondrogenic potential; AO with adipogenic and osteogenic potential; or AC with adipogenic and chondrogenic potential) or unipotent (O with osteogenic potential; or C with chondrogenic potential) clones analysed are shown, with their distribution in the upper and lower quartiles of:

(A) HUVEC total tubule length (TLL) following co-culture

(B) Osteogenic potential as measured by Alizarin Red staining assay,

(C) Adipogenic potential as measured by Oil Red O staining

**Fig. 1:**
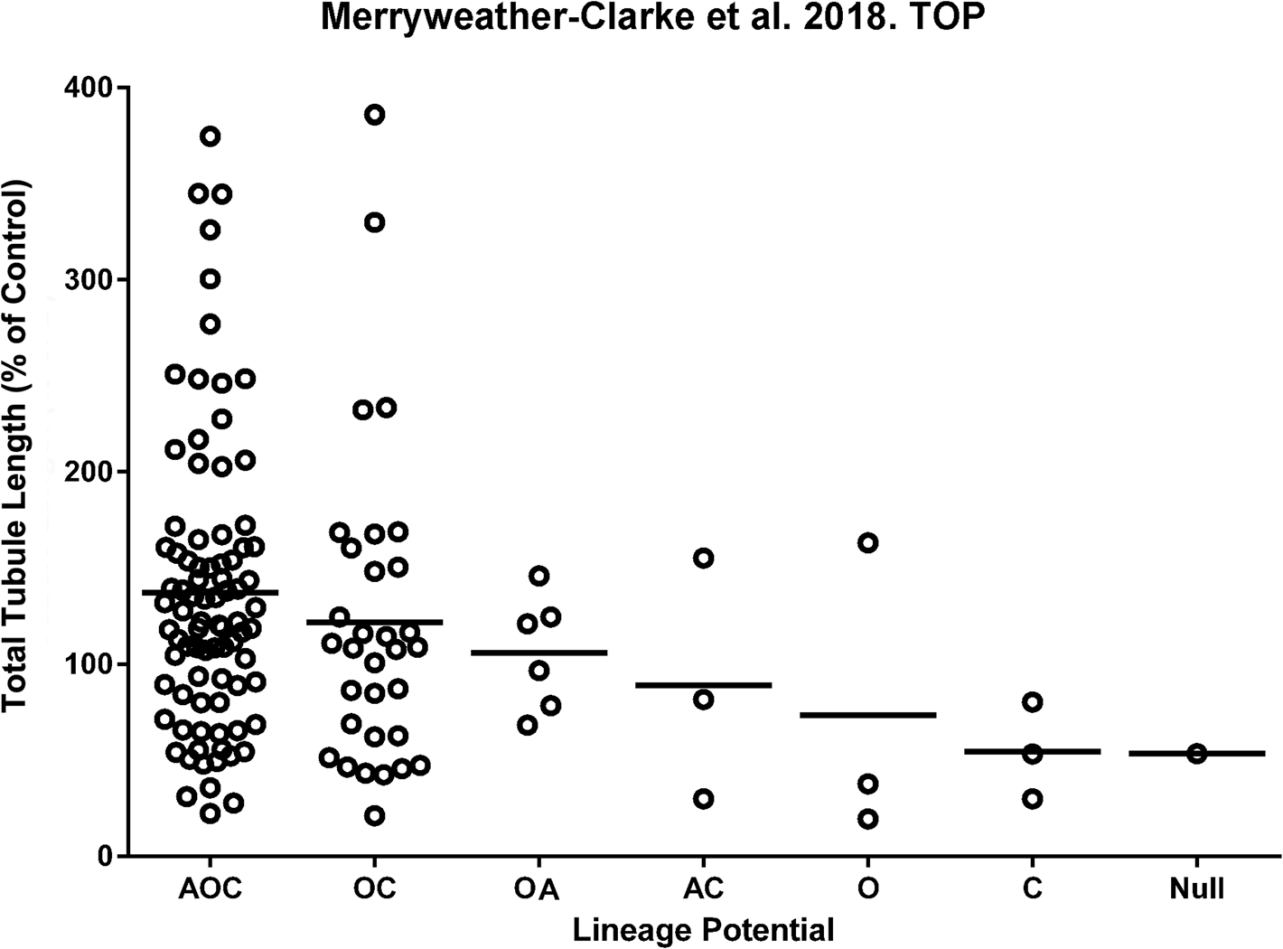
The relationship between lineage differentiation potential of CFU-F clones and their vascular tubule supportive capacity. A) Clonal cultures were categorised into groups based on their adipogenic (A), osteogenic (O) and chondrogenic (C) differentiation potential and this potency plotted against their ability to support day 14 vascular tubule formation in co-culture assays with HUVEC as measured by the total tubule length. The classification included tri-lineage (AOC), bi-lineage (OC, OA, AC), uni-lineage (O, C) and nullipotent (Null) clones. The total tubule length was normalised as a percentage of that obtained using a control non CFU-F selected hBM MSC sample (Control) which was set at 100%. Three bone marrow aspirates were used to generate 133 CFU-F clones. The bars represent the mean total tubule length (TTL) for each lineage subgroup. Quartiles for TTL are 0 to 68.67%, 68.67 to 116.6%, 116.6 to 156.3% and > 156.3%

The potency of the CFU-F clones to support vascular tubule formation by HUVEC varied when measured as total vascular tubule length in co-culture assays, ranging from very poor supporters generating few or no endothelial tubules, through to strong supporters, instigating long and highly branched networks of endothelial cells (Fig. 1; Additional file 1: Figure S1). Interestingly, the CFU-F with the highest mean vascular tubule supportive abilities shared osteogenic potential in common, regardless of whether they originated from the tri-lineage or bi-lineage clones (with trilineage {AOC} > bi-lineage {OC} > bi-lineage {OA} CFU-F clones; Table 2A; Fig. 1). The CFU-F clones that supported the strongest levels of vascular tubule formation were observed in the tri-lineage AOC and osteo-chondrogenic (OC) bi-lineage groups (Table 2A; Fig. 1), although it should be noted that most of the analysed clones exhibited these AOC and OC potentials making it difficult to assess the contribution of other clones with differing or more restrictive potency. These more potent vascular supportive AOC and OC CFU-F clones also had a higher capacity for osteogenesis as assessed quantitatively using the Alizarin Red assay (Table 2B). We therefore assessed the correlation between osteogenic differentiation potential and the effective support of the hBM MSCs on vascular network formation.

### Is there a correlation between CFU-F osteogenic and vascular supportive potency?

**Fig. 2:**
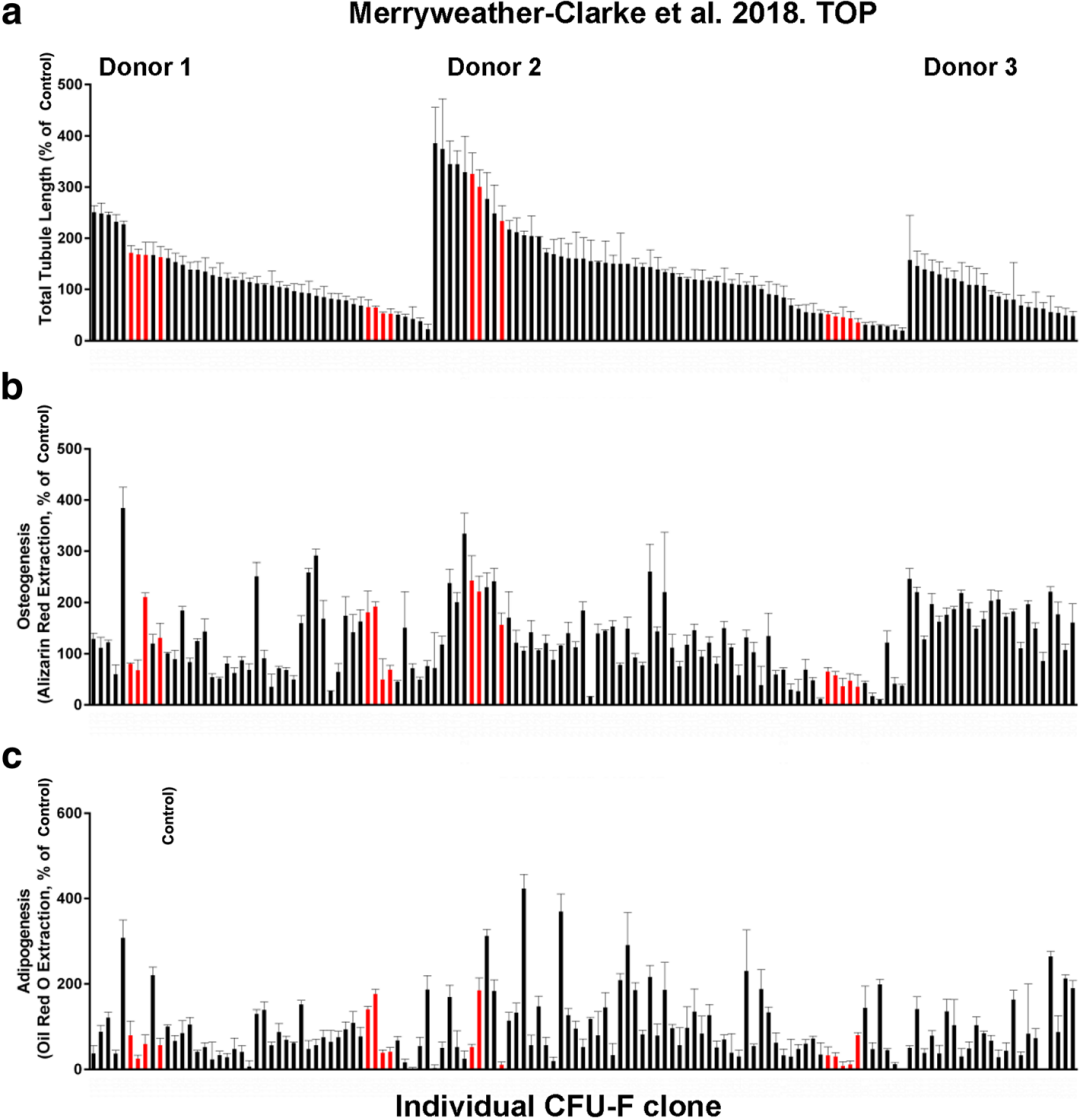
Quantification of vascular tubule supportive capacity, osteogenesis, and adipogenesis of hBM MSC clonal cultures. Clonal CFU-F cultures of hBM MSCs were expanded into T25 flasks before being assayed quantitatively at P1 for their osteogenic or adipogenic differentiation potential and their ability to support day 14 vascular tubule formation in co-culture assays with HUVEC as measured by the total tubule length. Three donor bone marrows were used (donor 1, donor 2 and donor 3). Data for individual CFU-F clones grouped by donor are shown. **a** HUVECs were seeded onto hBM MSC monolayers and cultured for 2 weeks before fixation and CD31 antibody staining. Total tubule length was calculated and normalised to the tubule length of a control hBM MSC sample (D), which was run for each separate experiment. The clonal cultures were assayed for their **b** osteogenic and **c** adipogenic differentiation potential by 2–3 weeks culture in differentiation media, relative to the control non CFU-F selected hBM MSC sample (Control). This control was set at 100% and all other values normalised against this. Values are mean ± SD of *n* = 3 replicate cultures. The histograms highlighted in red were used for cell sorting and RNAseq analyses

**Fig. 3:**
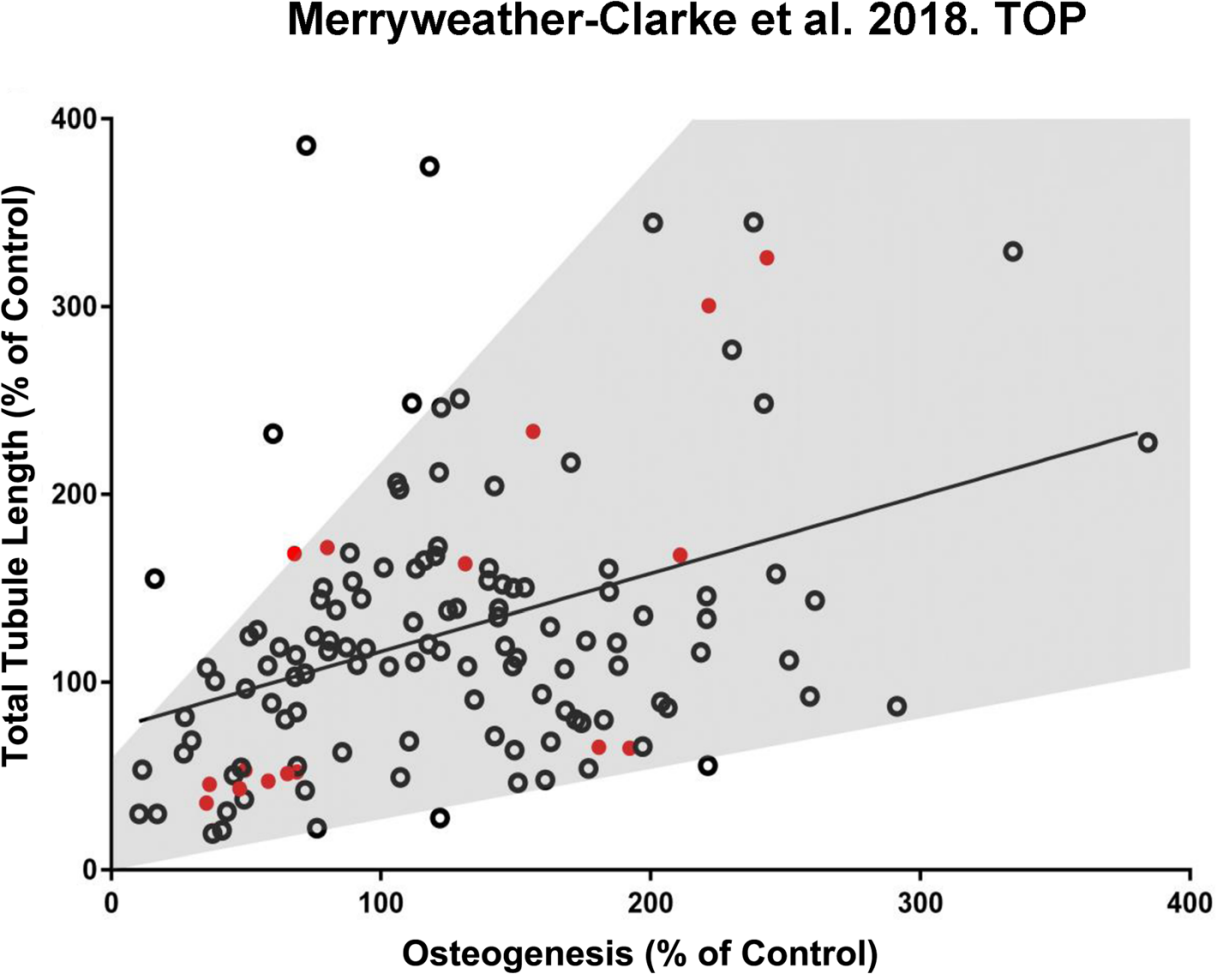
Correlations between osteogenic lineage differentiation potential and vascular tubule supportive capacity. Clonal hBM MSC CFU-F cultures at p1 were assayed quantitatively for their osteogenic differentiation potential after culture in osteogenic differentiation media, relative to the control non CFU-F selected hBM MSC sample (Control), which was set at 100%. and the correlation between osteogenic and vascular supportive activity assessed. Pearson’s correlation coefficient (*r*) was calculated for all three bone marrow donor aspirates. The red circles are CFU-F clones that were used for cell sorting and RNAseq analyses. There was a moderate positive relationship between the vascular tubule supportive function and the osteogenic potential for all CFU-F clones when these were assessed quantitatively (*p* [two tailed] < 0.0001; *n* = 133 clones)

The degree of osteogenesis and vascular tubule supportive activity, as well as adipogenesis, was quantified for all individual CFU-F clones at passage 1. The results in Fig. 2 demonstrate that these varied between the different clonal CFU-Fs when the three donor bone marrows were assessed together. Both donor variability and also CFU-F variability in terms of their functional potency in vitro were observed. When the osteogenic potential of all 133 CFU-F clones at passage 1 was quantitatively compared to their vascular tubule supportive capacity (Fig. 3), the strength of the association between these variables was significant with a Pearson’s correlation coefficient (*r*) of 0.3855 (*p* < 0.0001). Hence, there was a moderately positive relationship between increased osteogenesis and increased total tubule length in the co-culture assays (Fig. 3). The spread of the data was, however, relatively large, forming a cone like pattern (grey shaded area). Next, we compared, quantitatively, the vascular supportive potential with the osteogenic differentiation capacity of the CFU-F clones at passage 1 from the individual donors (Additional file 6: Figure S5). There was a moderately strong positive relationship between these variables for donors 2 and 3 (*r* = 0.6822, *p* value < 0.0001; *r* = 0.4125, *p* < 0.05 respectively). However, for donor 1, this relationship was poor (*r* = 0.1577; *p* (two tailed) = 0.2953). When the adipogenic potential of all CFU-F clones was quantitatively compared to their osteogenic or their vascular tubule supportive capacity at passage 1 (Additional file 7: Figure S6A and S6B respectively), the strength of the associations between these variables was low (respective *r* values of 0.2898 and 0.1523 with respective *p* values (two tailed) of < 0.001 and 0.0800). Notably, the correlation between adipogenic and vascular supportive potency of the CFU-F clones was not significantly different from zero.

### Transcriptome profiling of clonal CFU-F cultures by RNAseq

The findings described above suggest some degree of importance of either tri-lineage AOC or osteogenic, but not adipogenic, differentiation capacity in the ability of hBM MSCs to support endothelial tubule formation. However, due to the large degree of spread within the data, it is likely that other factors are also at play. These would include donor variability in CFU-F content and function. To discover gene fingerprints of the CFU-F clones which might predict these differences in potentiality, transcriptome profiling was performed using RNAseq. One hundred viable single cells from each of 16 CFU-F clones (that were good or poor supporters of vascular tubule networks as described in Table 2) were flow sorted and cDNA libraries generated using the method described [60]. All of these, 16 CFU-F-derived clones for RNAseq were characterised by osteogenic potential and vascular supportive activity being in either the upper or lower quartiles (red-coloured clones in Fig. 3).

**Fig. 4:**
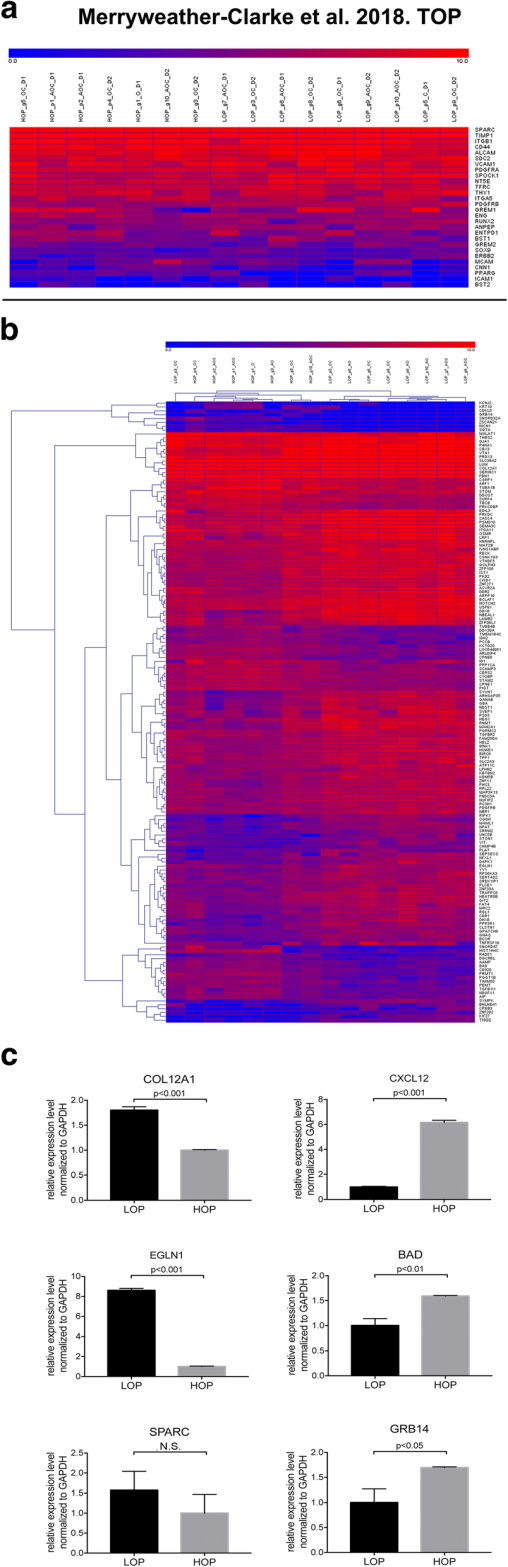
Heatmap of transcripts from RNA sequencing CFU-F clones with high versus low osteogenic differentiation capacity and qPCR analyses of candidate genes. **a** Well characterised hBM MSC genes detected by RNAseq. Expression level is indicated by the scale bar, as counts per million (CPM) mapped reads. Names of sequenced clones indicate whether they exhibited high or low osteogenic potential (HOP or LOP, respectively), good or poor vascular support (g or p), lineage potential (adipogenic (A), Osteogenic (O) and/or chondrogenic (C), and whether they originated from donor 1 (D1) or donor 2 (D2). **b** Hierarchical clustering of the 16 clones using 161 genes that were differentially expressed between HOP and LOP clones, determined using an FDR < 0.05. **c** qPCR analyses of COL12A1, EGLN1, CXCL12, BAD and GRB14 genes showing their statistically significant (< 0.05) up- or downregulaton in HOP versus LOP clones. SPARC represented a gene expressed in but not significantly differentially (N.S.) expressed between HOP and LOP clones. Details are described in the “Methods” section

We detected 11,698 transcripts expressed by the 16 selected CFU-F clones (Additional file 8: Table S2). Included amongst these are 28 well-characterised genes found in hBM MSCs (Fig. 4a). The results demonstrate consistently high levels of expression of some genes associated with hBM MSC longevity or quiescence (e.g. *SPARC/OSTEONECTIN*, *TIMP1*) and generally high, but slightly variable, levels of expression of MSC-associated adhesion or growth factor receptor transcripts (*ITGB1*, *CD44*, *ALCAM*, *SDC2/CD362*, *VCAM-1*, *PDGFRA*, *THY1/CD90*, *ITGB5*) across all 16 CFU-F clones. In contrast, other genes involved in skeletal formation and/or angiogenesis (e.g. *GREM1*, *RUNX2*, *SOX9*, *MCAM/CD146*, *PPARG*) were more variably or not as highly expressed across these 16 clones. This was particularly evident for master genes for chondrogenesis (*SOX9*) and adipogenesis (*PPARG*), when compared to osteogenesis (*RUNX2*). Genes expressed by the 16 clones at high levels (in the upper quartile range of expression magnitude), compared with genes expressed at levels in the lower quartile range, were enriched for many signalling pathways involving integrins and hypoxia-inducible factors (HIF; Additional file 9: Figure S7). The most significant of these were involved in integrin-mediated cell adhesion (*p* = 1.517E−10, FDR = 1.288E−08) and migration, and in the role of tetraspanins in integrin-mediated cell adhesion (*p* = 7.175E−09, FDR = 3.807E−07).

The 16 CFU-F-derived clones were also divided into different groups based on their tri-lineage, osteogenic, chondrogenic and adipogenic potential, and EdgeR used to explore the degree of differential expression dependent on each attribute. Osteogenic potential showed the most dynamic changes in gene expression with RNAseq (Fig. 4b). A total of 332 genes were differentially expressed in these 16 clones using an FDR < 0.1, while 161 genes were differentially expressed when determined using an FDR < 0.05 (Fig. 4b, Additional file 10: Table S3). The proangiogenic factor *CXCL12* (FDR = 0.086, 2.23 times upregulated in high osteogenic potential clones) was amongst the 332 genes differentially expressed at the lower stringency. Hierarchical clustering of the 16 clones using the 161 genes differentially expressed at the higher stringency broadly divides the clones into two clusters, low vs high osteogenic potential clones, with the exception of a single clone with low osteogenic potential which clusters with the high osteogenic potential clones (Fig. 4b). The 48 transcripts more highly expressed in clones with a higher osteogenic potential include growth factor receptor-bound protein 14 (*GRB14*, FDR = 0.038, 18.6-fold upregulated), BCL2-associated agonist of cell death (*BAD*, FDR = 0.039, threefold upregulated), transforming growth factor beta 1 induced transcript 1 (*TGFB1I1*, FDR = 0.019, 2.9-fold upregulated), the angiogenesis-associated long non-coding RNA *MALAT1* (FDR = 0.018, sevenfold upregulated), and the angio-associated migratory cell protein *AAMP* (FDR = 0.037, threefold upregulated). Ontology analysis using GeneCoDis [70] revealed that these 48 genes were significantly enriched for the VEGF signalling pathway (hypergeometric *p* value (Hyp) = 0.0028) and angiogenesis (Hyp = 0.0012). Genes more highly expressed in clones with lower osteogenic potential include *NOTCH2*, *EGLN1*, *TNFRSF19*, *UNC5B* and *COL12A1* (Fig. 4b; Additional file 10: Table S3) and were enriched for GO processes such as wound healing (*p* = 3.10E−04, FDR < 0.1), cardiovascular system development (*p* = 1.63E−10, FDR < 0.05), angiogenesis (*p* = 2.63E−05, FDR < 0.1) and bone remodelling (*p* = 4.72E−04, FDR < 0.05). qPCR analyses of five candidate genes (Fig. 4c) which were differentially expressed in higher (*CXCL12*, *BAD*, *GRB14*) versus lower (*COL12A1*, *EGLN1*) osteogenic potential CFU-F clones confirmed the differential expression of these genes observed in RNAseq. In terms of vascular supportive functions, tripotent AOC clones used for RNA sequencing include three good supporters of vascularisation and four poor supporters (Additional file 11: Figure S8). Of note, *TNFSF15* and *EPGN* are amongst ten genes differentially expressed (FDR < 0.05) between these two groups, being 16.4- and 1650-fold downregulated, while *LRIG2* was 12.9-fold upregulated, in AOC good, compared to poor, vascular supporters; these three genes are known to negatively or positively regulate blood vessel formation (Additional file 12: Table S4).

**Fig. 5:**
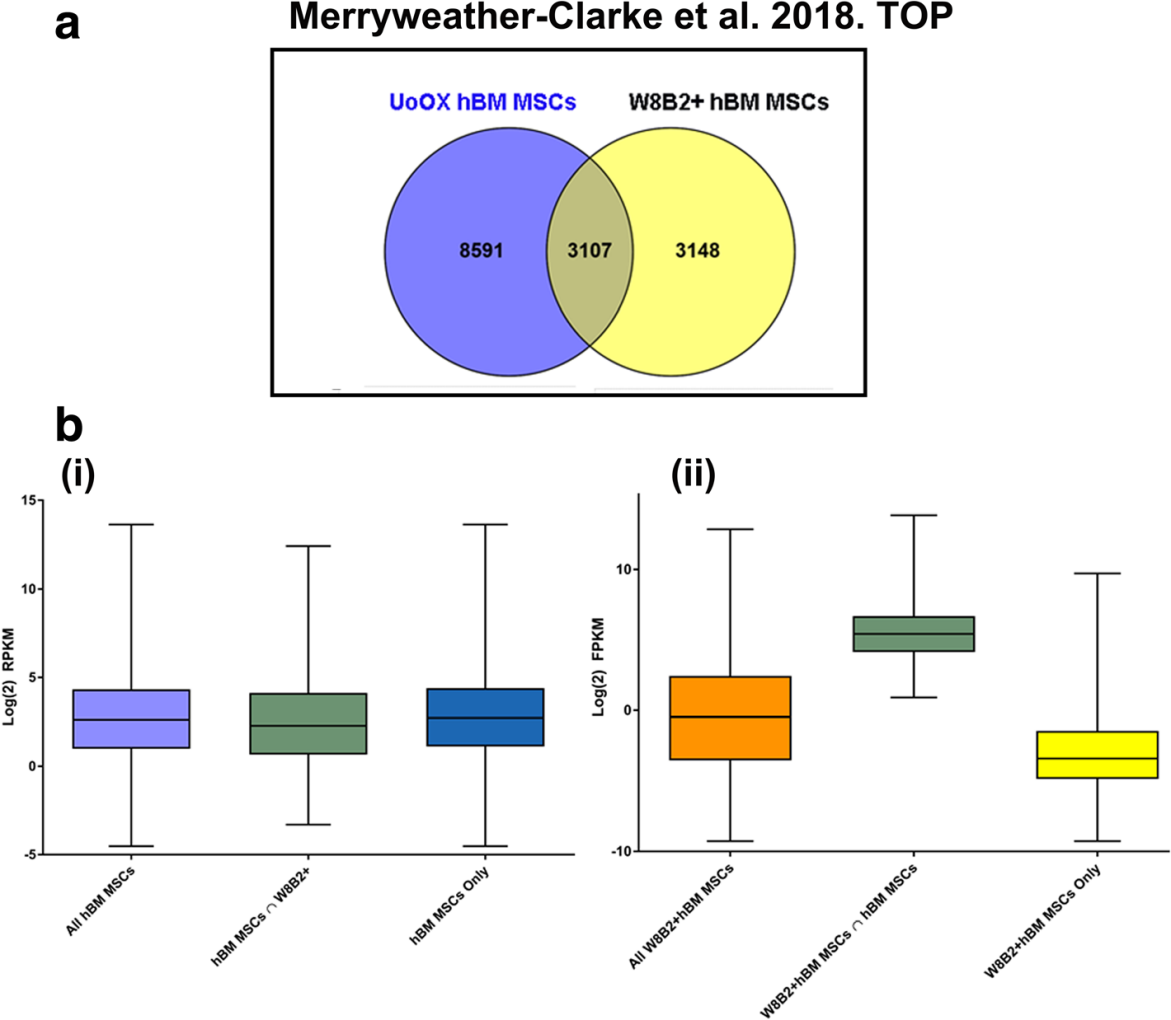
Comparison of hBM MSC RNA sequencing results with W8B2+ hBM MSC dataset. **a** Venn diagram showing the number of expressed genes shared between the datasets from this study (UoOX hBM MSCs) and from the published W8B2+ hBM MSCs data of Zhang et al. [68]. **b** Boxplots showing the expression levels of all genes, shared genes and unique genes in each dataset. **b** (i) gene expression levels in hBM MSCs from this study (UoOx hBM MSCs). **b** (ii) gene expression levels in W8B2+ hBM MSCs from the dataset of Zhang et al. [68]

The W8B2 antibody identifies mesenchymal stem cell antigen-1 (MSCA-1), which is restricted to CD271^bright^CD45^−^ hBM MSCs with a high CFU-F capacity [29]. Of 11,698 transcripts detected in our hBM MSCs (UoOX hBM MSC), 26.6% were also detected in a recently described primary W8B2+ enriched hBM MSC subset [67] (Fig. 5). The upper three quartiles of expression values of W8B2+ transcripts shared with our UoOX hBM MSCs are higher than those of transcripts unique to the W8B2+ subset (i.e. absent from our hBM MSCs; *p* < 0.0001, two-tailed *t* test, *t* = 144, df = 6523), demonstrating that transcripts expressed by our MSCs overlap with the more highly expressed transcripts detected by Zhang et al. [68]. Genes expressed by both sets of hBM MSCs include *HOX* transcription factor genes (*HOXA1*, *HOXA4*, *HOXA5*, *HOXA7*, *HOXA11*, *HOXB3*, *HOXB7*, *HOXC8*, *HOXC9*), as well as genes that regulate blood vessel formation, haematopoiesis, osteogenesis, chondrogenesis and adipogenesis (e.g. *ANGPTL4*, *ANGPTL5*, *BMP3*, *BMP4, EPGN, FZD1*, *FZD7*, *FGF2*, *GHR*, *GJA1*, *GRB14*, *IL7*, *IL15*, *JAG1*, *NOG*, *OMD*, *PCOLCE*, *PDGFD*, *PPARG*, *SFRP1*, *SPARC*, *SOX9*, *TGFB2*, *TGFB3*, *TNFRSF11B*, *TNFSF15*); some of these have been implicated in bone fracture repair.

## Discussion

The healing of bone fractures is complex, involving the inflammatory, repair and remodelling processes [71, 72]. The initial trauma with disruption of the blood supply is followed by the formation of a hematoma, which contains peripheral blood inflammatory cells and pro-inflammatory cytokines. With the release of pro-inflammatory and angiogenic factors, macrophage subsets and endothelial cells are attracted to the fracture site and then to the hematoma. The former cells then influence intramembranous bone formation and endochrondral ossification, while the latter cells drive new blood vessel formation and provide the associated pericytes or MSCs for osteogenic repair. These MSCs also suppress the inflammatory response [71, 72]. Delayed fracture healing can be caused by poor vascularisation and inadequate osteoprogenitor cell numbers, as elegantly reviewed in [73].

As new blood vessel formation is critical to bone fracture repair, we analysed, in this manuscript, the potential of clonally derived hBM MSC both to simultaneously support angio-/vasculo-genesis and for osteogenesis. We found that the majority of hBM MSC clones, which supported increased blood vessel formation in vitro were amongst those CFU-F-derived hBM MSCs which supported tri-lineage (AOC), and to a lesser extent bi-lineage (OC), differentiation. It is notable that the majority of the clones possessed these characteristics and hence it proved difficult to assess the contribution of other clones. However, the tri-lineage AOC clones at p1 were heterogeneous in their relative levels of vascular supportive potential. When we compared quantitatively the osteogenic or adipogenic differentiation potential of all the CFU-F-derived clones (whether they exhibited tripotent (AOC), bipotent (AO, AC or OC) or unipotent (O or A) potential) with their vascular tubule supportive capacity, we found a moderately strong correlation between increased osteogenesis and increased vascular supportive activity for two of three bone marrow donors examined. Thus, the correlation was donor dependent. There was no correlation between vascular supportive potential and increased adipogenesis of the CFU-F clones.

Our previous studies [73] have demonstrated that human cardiac-derived MSCs (termed cardiosphere-derived cells) from patients with ischemic heart disease also possess different donor-dependent levels of vascular supportive ability. In those studies, we showed that the potency of the cardiac-derived MSCs in reparative healing in an in vivo surrogate model of myocardial infarction was associated with their vascular supportive ability. This provides a consistent theme that assessing vascular supportive activity and, in this manuscript, also osteogenic potential, would very likely assist in identifying those patients who would benefit most from the use of autologous hBM MSC for reparative healing of large bone fractures and thus provides a basis for future studies. Why these differences in potency of hBM MSCs exist has not been fully explored. Likely mechanisms include both a genetic basis and environmental influences. Indeed, our recent studies suggest that environmental factors during pregnancy and early post-natal life can alter the molecular signature of cells leading to reduced blood vessel formation post-natally and increasing disease risk in later life [74, 75].

When we compared the transcriptomic profiles of 16 selected CFU-F clones with those found in the freshly isolated immature W8B2+ hBM MSC subset described by Zhang et al. [68], 26.6% of the expressed genes were shared. These included many genes with osteogenic (*SPARC/OSTEONECTIN*, *BMP3/OSTEOGENIN*, *BMP4*, *TNFRS11B/OSTEOPROTEGERIN*, *OMD/OSTEOMODULIN*, *FGF2*) and vascular (*ANGLT4*, *EPGN*, *FGF2*, *FZD7*, *FGF2*, *GHR*, *GJA1*, *JAG1*, *NOG*, *PDGD*, *SFRP1*, *TGFB2*, *TGFB3*, *TNFSF15*) regulatory functions. To better define the molecular signatures of clonal hBM MSC CFU-F, which display high osteogenic potential and also support high levels of new blood vessel formation, we further examined the RNAseq profiles of defined CFU-F clones at P1 with these characteristics.

A number of genes that regulate blood vessel formation were also differentially expressed in the CFU-F clones with high osteogenic and good vascular supportive activities in vitro. Interestingly, we observed upregulation of *CXCL12,* and enrichment for the VEGF signalling pathway (e.g. *BAD*, the BCL2-associated agonist of cell death involved in cell survival) in the CFU-F clones with high osteogenic potential. VEGF signalling not only couples osteogenesis to angiogenesis, but also controls osteoblast differentiation and function (reviewed in [72]). Notably, CXCL12 co-operates with VEGF to promote hBM MSC-induced new blood vessel formation [48, 76] by upregulating the CXCL12 receptor CXCR4 on endothelial cells. This serves to enhance vascular network formation by allowing endothelial tip-like cells to extend towards the CXCL12 guidance cue on adjacent endothelial cells or by promoting the integration of endothelial precursors (termed ECFC) into the developing vascular networks [48]. We also found that the lnc-RNA, *MALAT1* (metastasis-associated lung adenocarcinoma transcript 1) was enriched in hBM MSC clones with high osteogenic potential. This lnc-RNA has an important role in osteogenic differentiation, promoting bone formation and mineralisation [77]. *MALAT1* also promotes the proliferation of human endothelial stalk cells and suppresses endothelial migratory activity [78]. Since *MALAT1* has been reported to promote cancer-induced angiogenesis through exosomal transfer from stromal cells to other cells in the tumour environment [79], we might speculate that a similar mechanism occurs in bone regeneration between hBM MSCs with high osteoblastic potential and endothelial cells, thereby communicating the need for and promoting enhanced vessel formation during bone fracture repair. This is an area for further investigation and beyond the scope of this current manuscript. Additionally, *LRIG2* (leucine-rich repeats and immunoglobulin-like domains 2) was upregulated in those AOC tri-lineage CFU-F clones with good vascular supportive activity. This molecule has been reported to enhance human vascular endothelial cell migration and tubule formation, a mechanism which may involve the VEGF signalling pathway [80].

In contrast to CFU-F clones with high osteoblastic potential, a number of genes were upregulated in CFU-F clones with low osteogenic potential. These include (i) *NOTCH2* (twofold upregulated; *p* = 5.57E−04, FDR = 0.047), which plays a predominant role in suppressing osteoblastogenesis [81]; (ii) *COL12A1* (3.49-fold upregulated; *p* = 1.33E−06, FDR = 1.94E−03), a collagen-encoding gene that forms complexes mediating osteoblast interactions during osteogenesis [82]; (iii) *UNC5B* (4.51-fold upregulated; *p* = 4.00E−05, FDR = 1.57E−02), which encodes a netrin receptor involved in negative regulation of osteoblast differentiation by netrin 1 [83, 84]; (iv) *TNFRSF19* (4.38-fold upregulated; *p* = 6.75E−04; FDR = 4.92E−02), which encodes a factor that regulates human MSC differentiation into osteoblasts or adipocytes [85]; and (v) *EGLN1*, (2.49-fold upregulated; *p* = 4.02E−05; FDR = 0.016) which encodes PHD2 (propyl hydroxylase domain 2 protein), a cellular oxygen sensor involved in HIF-α proteosomal degradation in normoxic conditions and inhibiting osteogenic differentiation of MSCs and their upregulation of proangiogenic factors [86–88]. Additionally, in those CFU-F with poor vascular supportive capacity, there was an upregulation of *TNFSF15* (VEGI), a potent inhibitor of endothelial proliferation and angiogenesis [89]. Thus, the cellular potential and RNA sequencing evidence presented in this manuscript support the view that there is some degree of importance of either tri-lineage AOC or osteogenic, but not adipogenic, differentiation capacity in the ability of hBM MSCs to support endothelial tubule formation. However, it is likely that other factors (including donor variability in CFU-F content and function) also come into play. These will likely become the focus of future research efforts that will aid the translation of these cells into optimal clinical practice.

## Conclusion

In conclusion, we have identified a moderately strong correlation between osteogenic and vascular supportive potential of CFU-F clones at p1 but show that this is donor dependent. Furthermore, we have defined important gene signatures which characterise the potential of these clones to promote osteogenesis and new blood vessel formation, processes required for enhancing the repair of significant bone fractures in difficult to treat patients.

## Additional files

Additional file 1: **Figure S1.** Examples of assays used to assess potency of CFU-F clonal cultures at P1. Left panel: Examples of high (left), intermediate (middle) and poor (right) levels of adipogenic, chondrogenic, osteogenic and vascular supportive capacity of CFU-F clones at P1. Right panel: Examples of positive (left) versus negative (right) control cultures (Control non CFU-F selected hBM MSC) for adipogenic, chondrogenic and osteogenic differentiation capacity. Also shown is an example of vascular tubule growth by HUVECs used as the positive control for standardisation between experiments. (TIF 2171 kb)

Additional file 2: **Table S1.** Phenotype of hBM MSCs p1. (DOCX 14 kb)

Additional file 3: **Figure S2.** CFU-F clonal assay. a and b) show typical morphology of CFU-F clones in culture at P1. c-e) show CFU-F clones from human bone marrow aspirates at D14, each set from the 3 different donors. (TIF 1206 kb)

Additional file 4: **Figure S3.** CFU-F morphologies at P1. Shown is the spindle like fibroblastoid morphology for 24 individual CFU-F clones at P1 from bone marrow donor 1. (TIF 4276 kb)

Additional file 5: **Figure S4.** CFU-F morphologies at P1. Shown is the spindle like fibroblastoid morphology for 28 individual CFU-F clones at P1 from bone marrow donor 2. (TIF 4980 kb)

Additional file 6: **Figure S5.** Correlations between osteogenic lineage differentiation potential and vascular tubule supportive capacity. Clonal hBM MSC CFU-F cultures at p1 were assayed quantitatively for their osteogenic differentiation potential after culture in osteogenic differentiation media, relative to the control non CFU-F selected hBM MSC sample (Control), which was set at 100%, and the correlation between osteogenic and vascular supportive activity assessed. A to C) Pearson’s correlation coefficient (*r*) was calculated for individually for each donor bone marrow aspirate (donor 1–3 respectively). The strongest positive relationship between the vascular tubule supportive function and the osteogenic potential was for CFU-F clones from donor 2 (B) when these were assessed quantitatively (p [2 tailed] < 0.0001; *n* = 63 clones). (TIF 466 kb)

Additional file 7: **Figure S6.** Correlations between adipogenic lineage differentiation potential with osteogenic or vascular tubule supportive capacity. Clonal hBM MSC CFU-F cultures at p1 were assayed quantitatively for their adipogenic, osteogenic or vascular supportive potential after culture in specific differentiation media or assays, relative to the control non CFU-F selected hBM MSC sample (Control), which was set at 100%. Correlations were determined using Pearson’s correlation coefficient (*r*) for the 3 bone marrow donor aspirates between A) osteogenic and adipogenic lineage differentiation potential and B) adipogenic versus vascular supportive capacity for the 3 donor bone marrows tested. Both show poor correlations between osteogenic vs adipogenic potential and adipogenic vs vascular supportive activity. (TIF 6791 kb)

Additional file 8: **Table S2.** Reads Per Kilobase of transcript per Million mapped reads (RPKM) for the sixteen sequenced clones. (XLSX 2919 kb)

Additional file 9: **Figure S7.** Pathways enriched in genes in the upper expression quartile versus the lower expression quartile. The negative log of the *p* value returned by Metacore for association of genes with pathways. Red, upper quartile (Metacore objects exclusively associated with the most highly expressed genes); Blue, lower quartile (Metacore objects exclusively associated with the least highly expressed genes). Purple, Metacore objects in common between the two sets of genes. (TIF 774 kb)

Additional file 10: **Table S3.** Genes differentially Expressed between clones with high osteogenic potential (HOP) and those with low osteogenic potential (LOP). (DOCX 81 kb)

Additional file 11: **Figure S8.** CFU-F clones with AOC tri-lineage differentiation potential and differing vascular tubule supportive capacity selected for RNA sequencing. Clonal cultures from 3 different bone marrow donors were categorised into groups based on their AOC differentiation potential and this potency plotted against their ability to support day 14 vascular tubule formation in co-culture assays with HUVEC as measured by the total tubule length. The total tubule length was normalised as a percentage of that obtained using a control non CFU-F selected hBM MSC sample (Control) which was set at 100%. The bar represents the mean total tubule length (TTL) for each lineage subgroup. The red coloured dots were clones from the AOC subset selected for sorting and RNA sequencing. (TIF 205 kb)

Additional file 12: **Table S4.** Genes differentially expressed between good and poor vascular supportive CFU-F clones. (DOCX 285 kb)

## Abbreviations

A: Adipogenic
BSA: Bovine serum albumin
C: Chondrogenic
CFU-F: Colony forming unit-fibroblastoid
DAPI: 4′,6-Diamidino-2-phenylindole, dihydrochloride
EGM2: Endothelial growth medium 2
FACS: Fluorescence-activated cell sorter
FDR: False discovery rate
hBM MSCs: Human bone marrow mesenchymal stem/stromal cells
HUVEC: Human umbilical vein endothelial cell
MSCGM: Mesenchymal stem/stromal cell growth medium
MSCs: Mesenchymal stem/stromal cells
MSPCs: Mesenchymal stem and progenitor cells
O: Osteogenic
P1: Passage 1
PBS: Phosphate-buffered saline
PFA: Paraformaldehyde
RNAseq: RNA sequencing
SSCs: Skeletal stem cells
TTL: Total tubule length
UoOx: University of Oxford
