# Supplementary figures and images for: Does osteogenic potential of clonal human bone marrow mesenchymal stem/stromal cells correlate with their vascular supportive ability?
Source: Stem Cell Res Ther. 2018 Dec 19;9:351. doi: 10.1186/s13287-018-1095-7 (PMC6300038; doi:10.1186/s13287-018-1095-7)

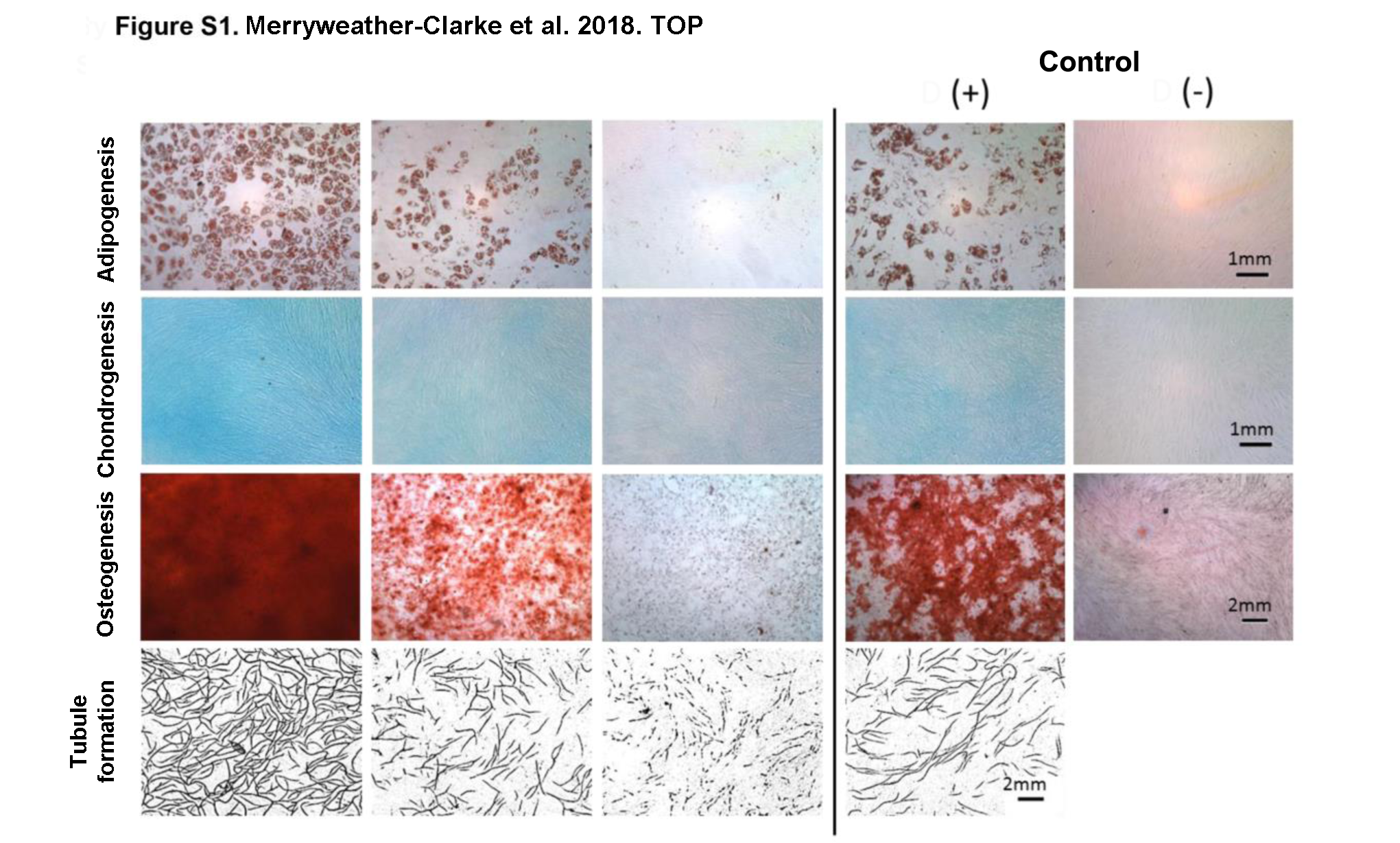

Supplement: Supplementary file 1 — Figure S1. Examples of assays used to assess potency of CFU-F clonal cultures at P1. Left panel: Examples of high (left), intermediate (middle) and poor (right) levels of adipogenic, chondrogenic, osteogenic and vascular supportive capacity of CFU-F clones at P1. Right panel: Examples of positive (left) versus negative (right) control cultures (Control non CFU-F selected hBM MSC) for adipogenic, chondrogenic and osteogenic differentiation capacity. Also shown is an example of vascular tubule growth by HUVECs used as the positive control for standardisation between experiments. (TIF 2171 kb) [file 13287_2018_1095_MOESM1_ESM.tif]

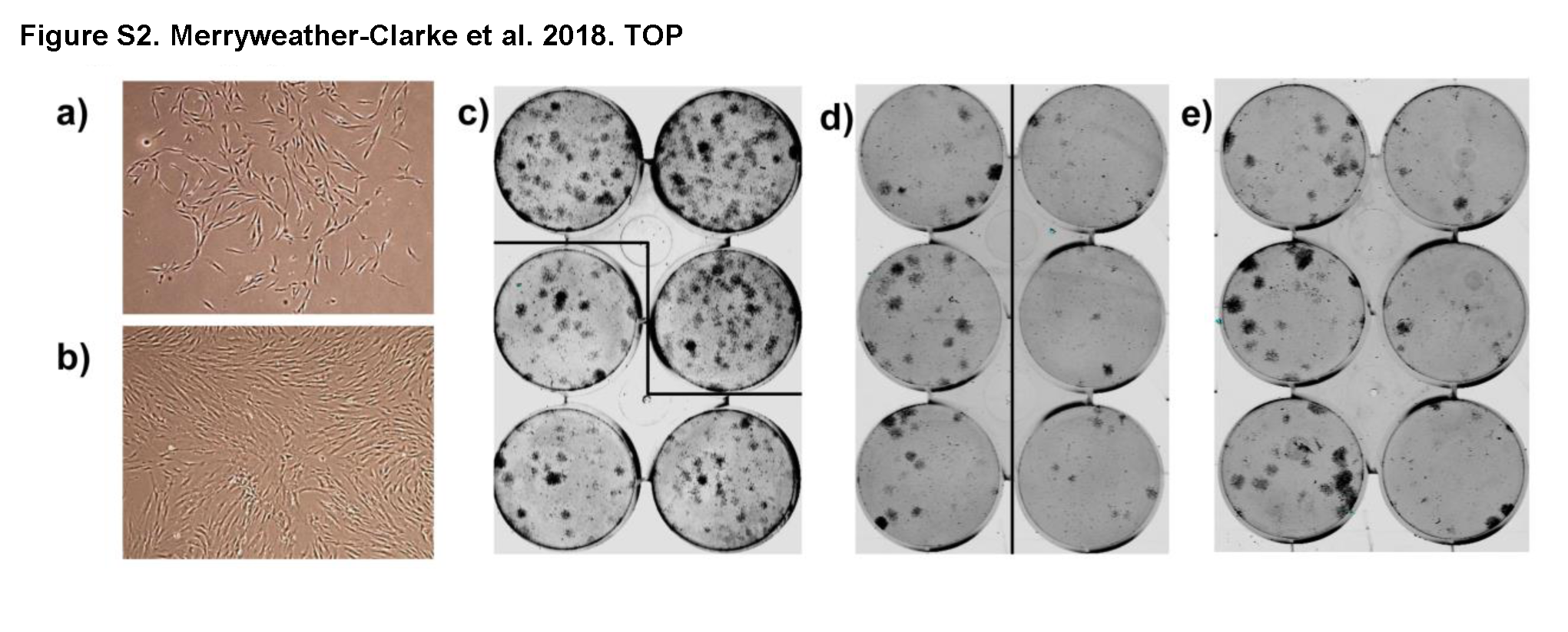

Supplement: Supplementary file 3 — Figure S2. CFU-F clonal assay. a and b) show typical morphology of CFU-F clones in culture at P1. c-e) show CFU-F clones from human bone marrow aspirates at D14, each set from the 3 different donors. (TIF 1206 kb) [file 13287_2018_1095_MOESM3_ESM.tif]

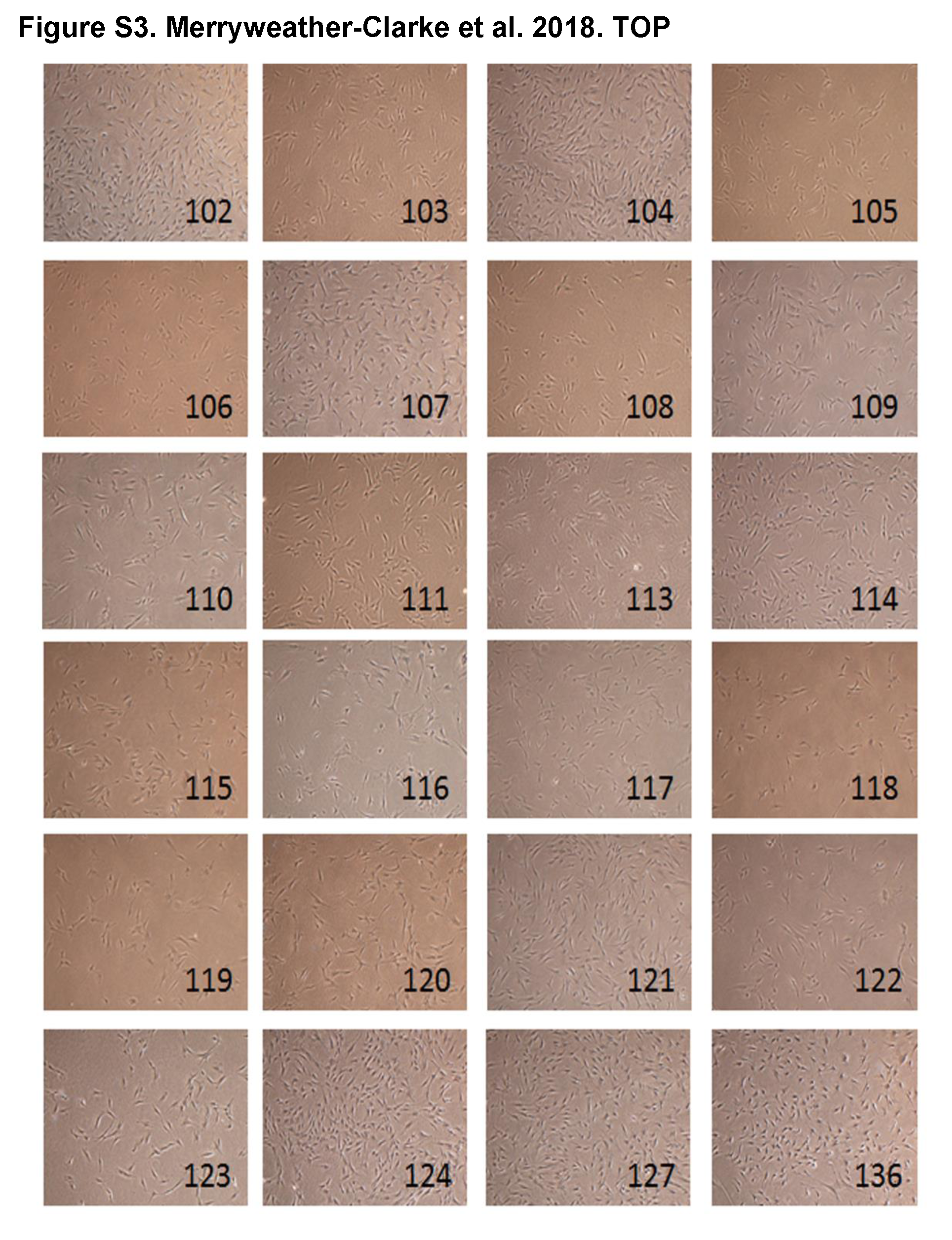

Supplement: Supplementary file 4 — Figure S3. CFU-F morphologies at P1. Shown is the spindle like fibroblastoid morphology for 24 individual CFU-F clones at P1 from bone marrow donor 1. (TIF 4276 kb) [file 13287_2018_1095_MOESM4_ESM.tif]

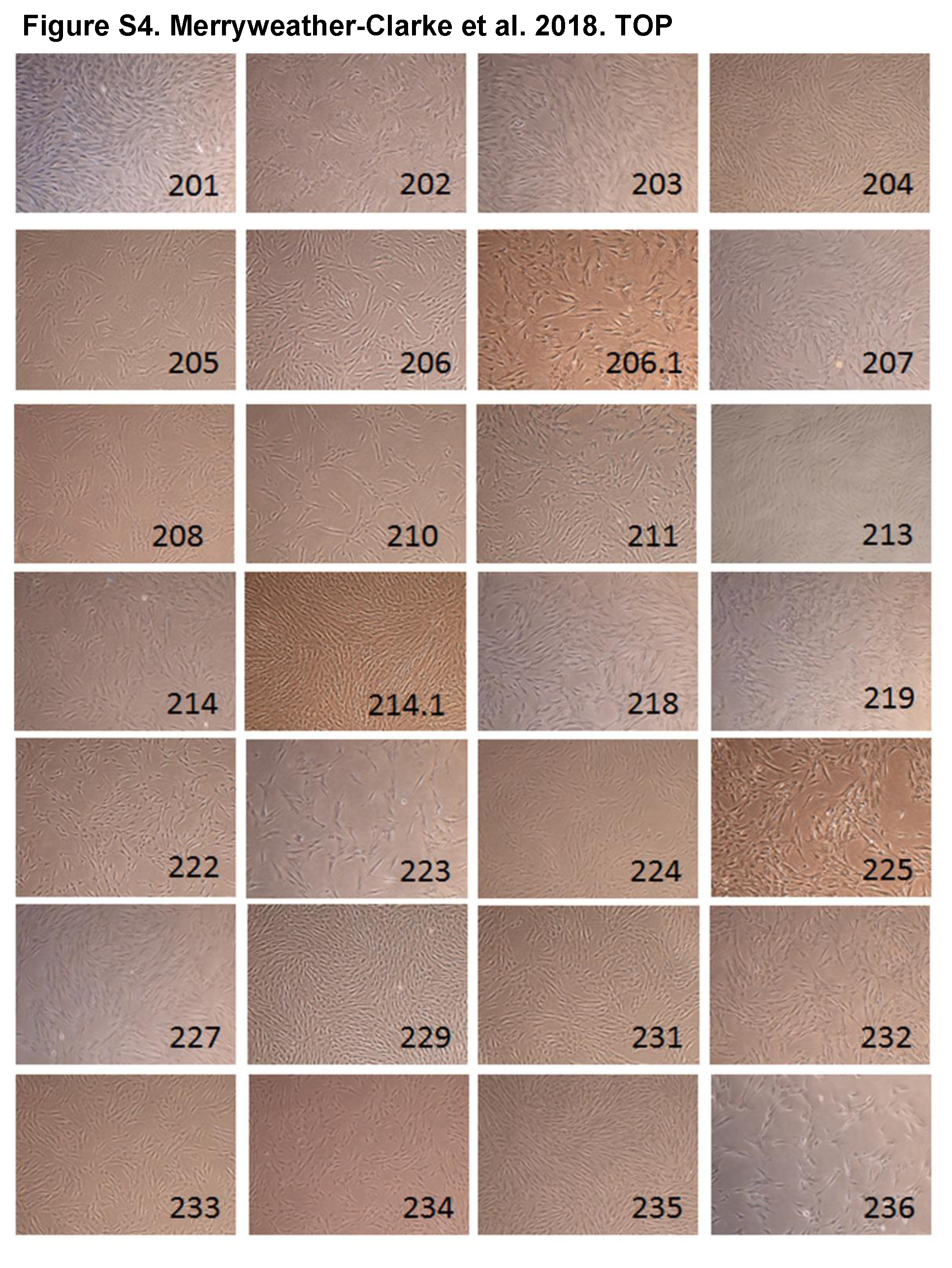

Supplement: Supplementary file 5 — Figure S4. CFU-F morphologies at P1. Shown is the spindle like fibroblastoid morphology for 28 individual CFU-F clones at P1 from bone marrow donor 2. (TIF 4980 kb) [file 13287_2018_1095_MOESM5_ESM.tif]

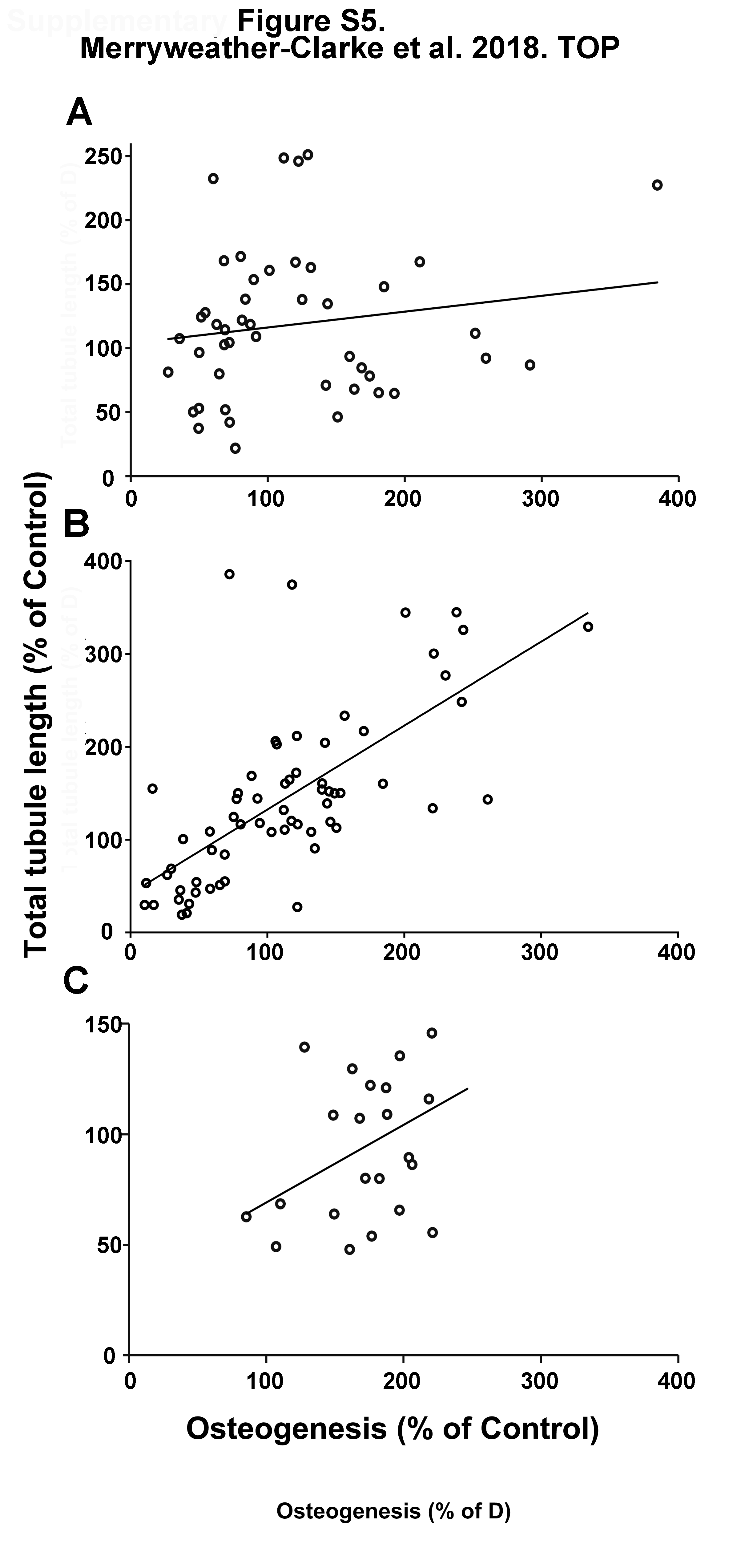

Supplement: Supplementary file 6 — Figure S5. Correlations between osteogenic lineage differentiation potential and vascular tubule supportive capacity. Clonal hBM MSC CFU-F cultures at p1 were assayed quantitatively for their osteogenic differentiation potential after culture in osteogenic differentiation media, relative to the control non CFU-F selected hBM MSC sample (Control), which was set at 100%, and the correlation between osteogenic and vascular supportive activity assessed. A to C) Pearson’s correlation coefficient (r) was calculated for individually for each donor bone marrow aspirate (donor 1–3 respectively). The strongest positive relationship between the vascular tubule supportive function and the osteogenic potential was for CFU-F clones from donor 2 (B) when these were assessed quantitatively (p [2 tailed] < 0.0001; n = 63 clones). (TIF 466 kb) [file 13287_2018_1095_MOESM6_ESM.tif]

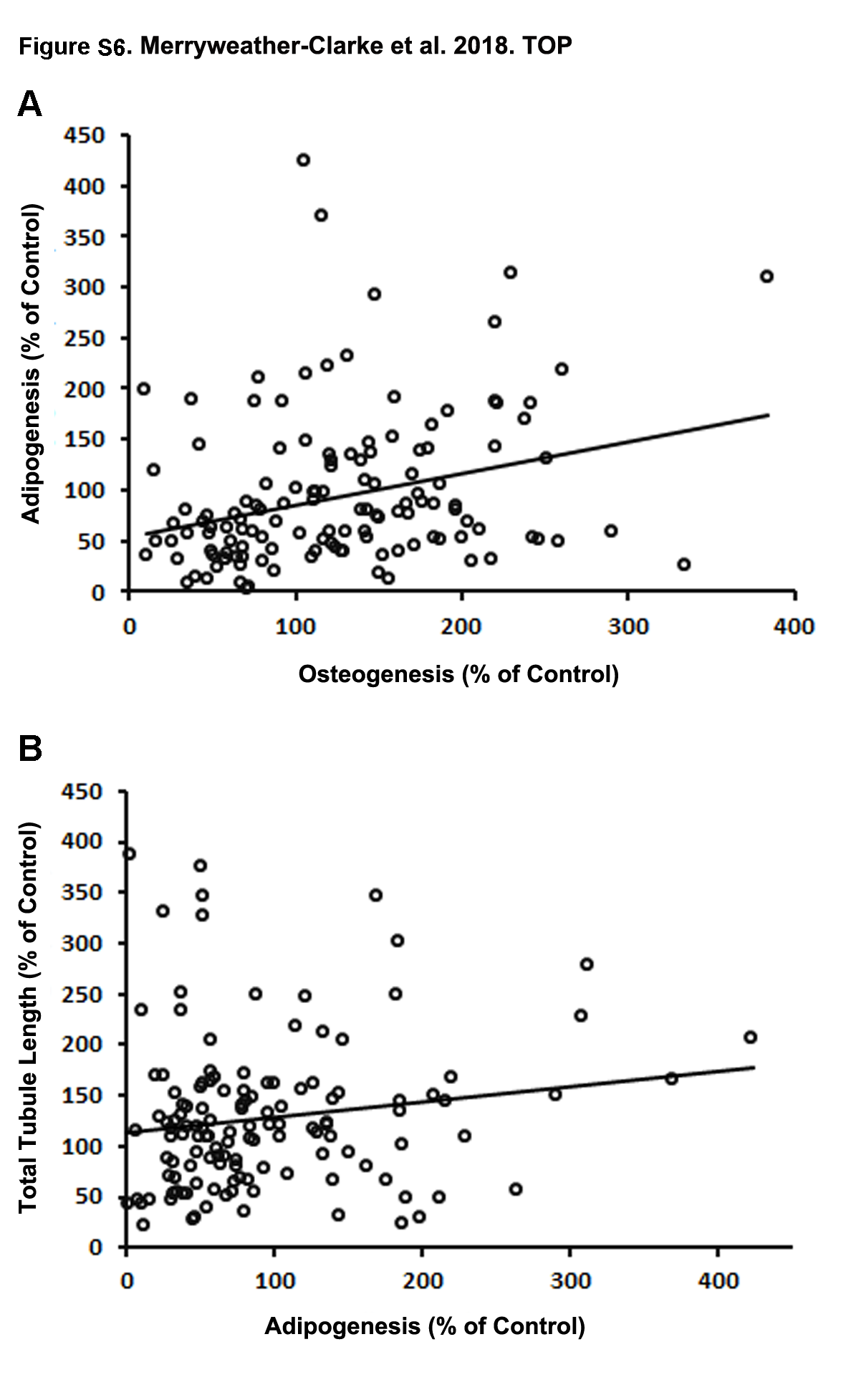

Supplement: Supplementary file 7 — Figure S6. Correlations between adipogenic lineage differentiation potential with osteogenic or vascular tubule supportive capacity. Clonal hBM MSC CFU-F cultures at p1 were assayed quantitatively for their adipogenic, osteogenic or vascular supportive potential after culture in specific differentiation media or assays, relative to the control non CFU-F selected hBM MSC sample (Control), which was set at 100%. Correlations were determined using Pearson’s correlation coefficient (r) for the 3 bone marrow donor aspirates between A) osteogenic and adipogenic lineage differentiation potential and B) adipogenic versus vascular supportive capacity for the 3 donor bone marrows tested. Both show poor correlations between osteogenic vs adipogenic potential and adipogenic vs vascular supportive activity. (TIF 6791 kb) [file 13287_2018_1095_MOESM7_ESM.tif]

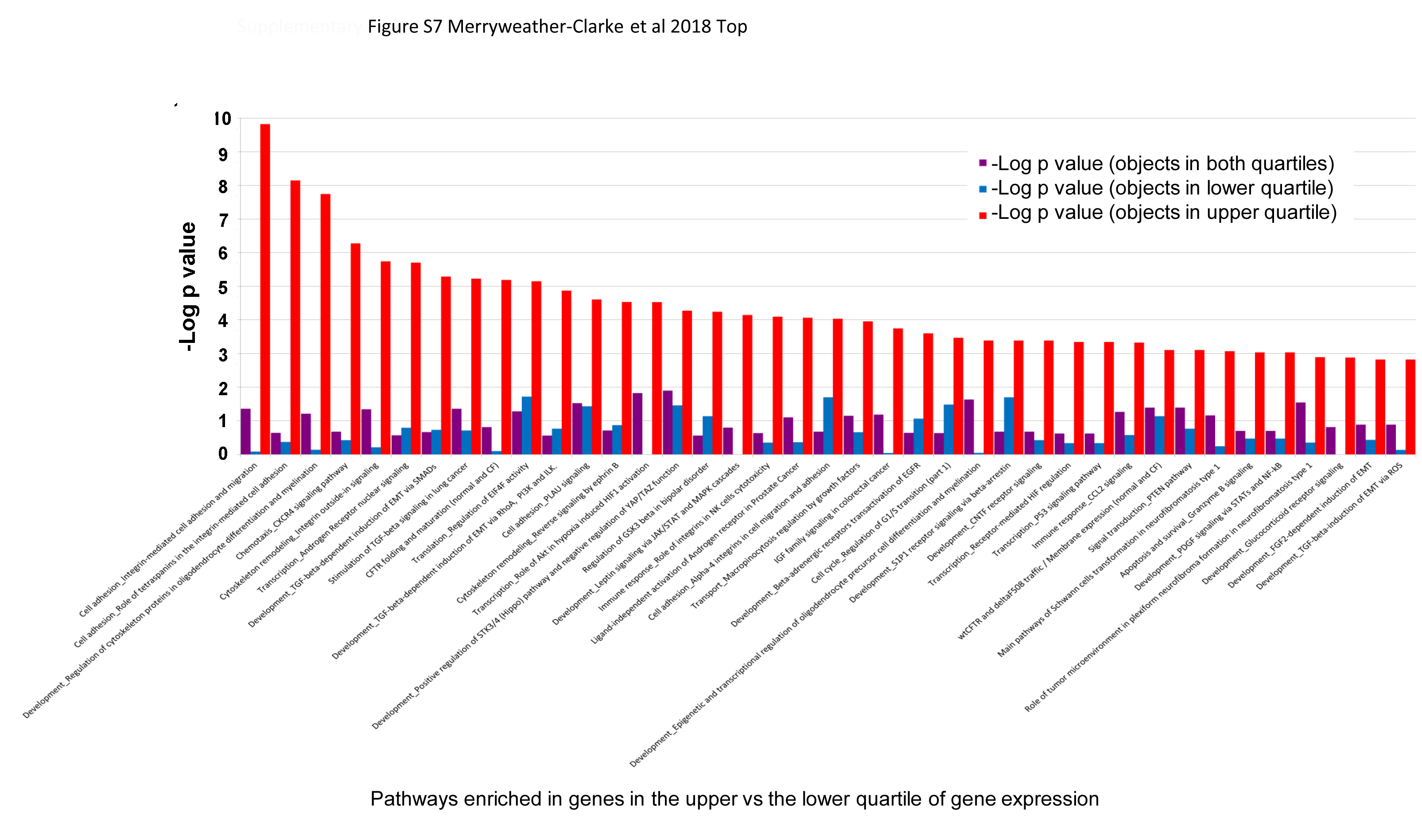

Supplement: Supplementary file 9 — Figure S7. Pathways enriched in genes in the upper expression quartile versus the lower expression quartile. The negative log of the p value returned by Metacore for association of genes with pathways. Red, upper quartile (Metacore objects exclusively associated with the most highly expressed genes); Blue, lower quartile (Metacore objects exclusively associated with the least highly expressed genes). Purple, Metacore objects in common between the two sets of genes. (TIF 774 kb) [file 13287_2018_1095_MOESM9_ESM.tif]
