# Supplementary material for: Does osteogenic potential of clonal human bone marrow mesenchymal stem/stromal cells correlate with their vascular supportive ability?
Source: Stem Cell Res Ther. 2018 Dec 19;9:351. doi: 10.1186/s13287-018-1095-7 (PMC6300038; doi:10.1186/s13287-018-1095-7)
Supplement: Supplementary file 2 — Table S1. Phenotype of hBM MSCs p1. (DOCX 14 kb) [file 13287_2018_1095_MOESM2_ESM.docx]

**Table S1: Phenotype of hBM MSCs p1**

| **Antibody** | **Antibody Source**  **(Murine)** | **MFI**  **(isotype control)**  **Mean+SD** | **MFI**  **(hBM MSCs)**  **Mean+SD** | **Result** |
| --- | --- | --- | --- | --- |
| CD45-PerCP | BD Biosciences | 8.8 + 6.0 | 8.6 + 3.4 | Negative |
| CD90-FITC | BD Biosciences | 8.8 + 6.0 | 80.2 + 39.3 | Positive |
| CD105-FITC | R&D Systems | 8.8 + 6.0 | 80.2 + 38.0 | Positive |
| CD73-PE | BD Biosciences | 7.3 + 5.6 | 159.5 + 29.0 | Positive |
| CD166-PE | R&D Systems | 7.3 + 5.6 | 789.7 + 228.0 | Positive |
| CD146-APC | Millipore (Chemicon) | 140.1 + 111.6 | 403.3 + 159.9 | Positive |

Values are mean + SD of n=3 bone marrow.

Cells were also stained with an APC-labeled rat anti-CD362 antibody from Lifespan-Biosystems and were positive (MFI = 755+361) compared to the negative control (MFI = 58+66).
