# Supplementary material for: Does osteogenic potential of clonal human bone marrow mesenchymal stem/stromal cells correlate with their vascular supportive ability?
Source: Stem Cell Res Ther. 2018 Dec 19;9:351. doi: 10.1186/s13287-018-1095-7 (PMC6300038; doi:10.1186/s13287-018-1095-7)
Supplement: Supplementary file 10 — Table S3. Genes differentially Expressed between clones with high osteogenic potential (HOP) and those with low osteogenic potential (LOP). (DOCX 81 kb) [file 13287_2018_1095_MOESM10_ESM.docx]

**Supplementary Table S3**

Genes differentially Expressed between clones with high osteogenic potential (HOP) and those with low osteogenic potential

(LOP).

Log and Linear (Lin) Fold Change (FC), p value, False Discovery Rate (FDR) and average log counts per million (AvLogCPM) are

shown.

S3A, genes up-regulated in clones with HOP.

S3B, genes down-regulated in clones with HOP.

Supplementary Table S3A

Supplementary Table S3B
