# Supplementary material for: Does osteogenic potential of clonal human bone marrow mesenchymal stem/stromal cells correlate with their vascular supportive ability?
Source: Stem Cell Res Ther. 2018 Dec 19;9:351. doi: 10.1186/s13287-018-1095-7 (PMC6300038; doi:10.1186/s13287-018-1095-7)

**Supplementary Table S4**

Genes differentially expressed between three tripotent clones that were good supporters of vascularisation (g7, g9,

g10; AOC3Gs) and four tripotent clones that were poor supporters of vascularisation (p1, p2, p6, p10; AOC4Ps).

Log Fold Change (LogFC), False Discovery Rate (FDR) and average log counts per million (AvLogCPM) are shown for the

three good supporters and four poor supporters of vascularisation.


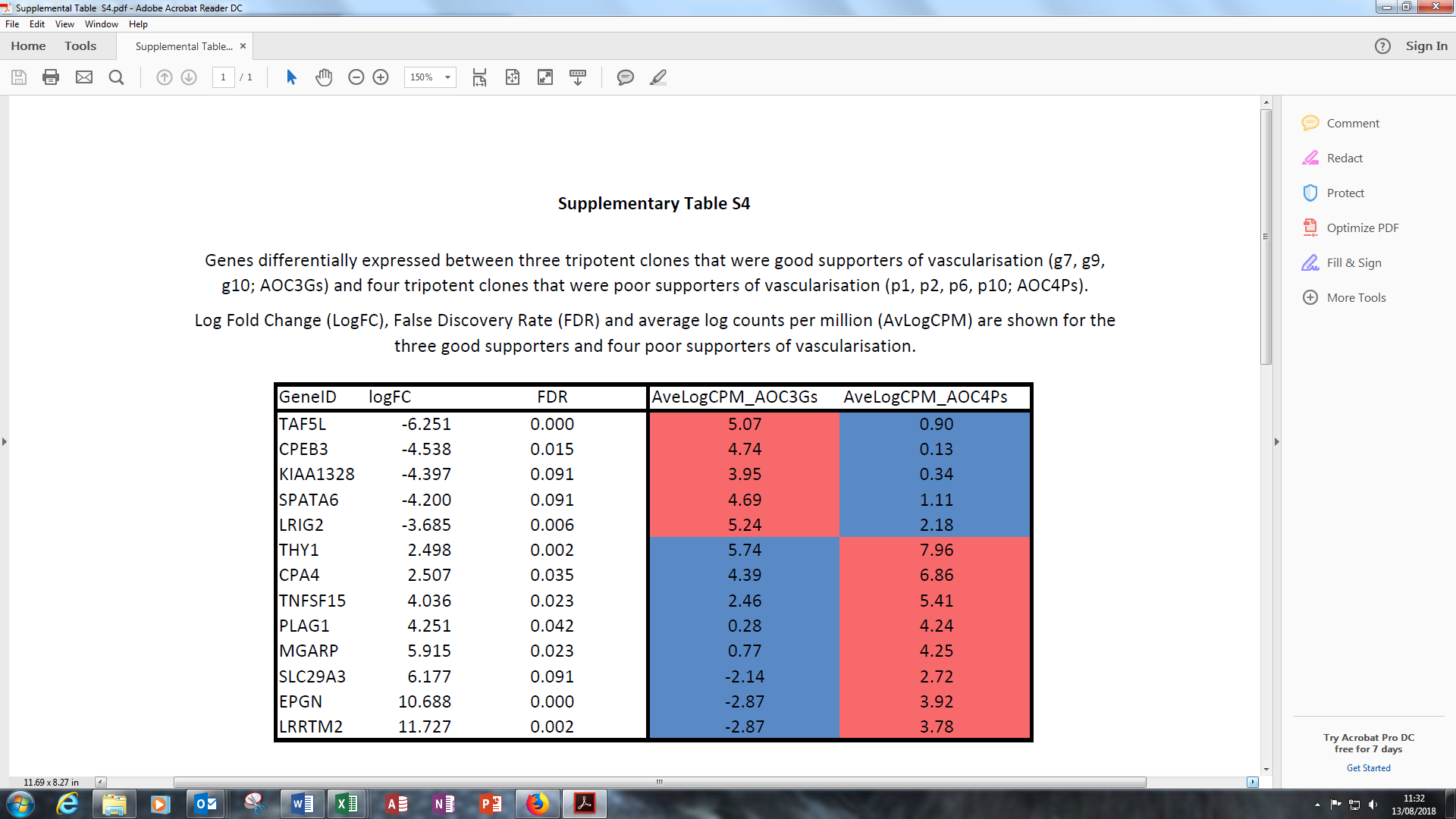

Supplement: Supplementary file 12 — Table S4. Genes differentially expressed between good and poor vascular supportive CFU-F clones. (DOCX 285 kb) [file 13287_2018_1095_MOESM12_ESM.docx]
